# Luteal phase support for in vitro fertilization/intracytoplasmic sperm injection fresh cycles: a systematic review and network meta-analysis

**DOI:** 10.1186/s12958-021-00782-5

**Published:** 2021-07-06

**Authors:** Hanglin Wu, Songying Zhang, Xiaona Lin, Shasha Wang, Ping Zhou

**Affiliations:** 1 Department of Obstetrics and Gynaecology, Hangzhou Women’s Hospital, No. 369 Kun Peng Road, Hangzhou, 310008 Zhejiang China; 2 Assisted Reproduction Unit, Department of Obstetrics and Gynecology, Sir Run Run Shaw Hospital, Zhejiang University School of Medicine, No. 3 Qingchun East Road, Hangzhou, 310016 China

**Keywords:** In vitro fertilization, Intracytoplasmic sperm injection, Luteal phase supports, Pregnancy rate, Network meta-analysis

## Abstract

**Background:**

Various luteal phase supports (LPSs) have been proven to increase the pregnancy rate in fresh cycles of in vitro fertilization or intracytoplasmic sperm injection; however, there is still significant debate regarding the optimal use of LPS.

**Methods:**

A systematic review with the use of a network meta-analysis was performed via electronic searching of Ovid MEDLINE, the Cochrane Library, Embase, Web of Science, ClinicalTrials.gov and Google Scholar (up to January 2021) to compare the effectiveness and safety of various LPSs, as well as to evaluate the effects of different initiations of LPSs on pregnancy outcomes. The primary outcomes included live birth and ongoing pregnancy, with the results presented as odds ratios (ORs) with 95% confidence intervals (CIs).

**Results:**

Eighty-nine randomized controlled trials with 29,625 women comparing 14 interventions or placebo/no LPS treatments were included in the meta-analyses. No significant differences were found in terms of the pregnancy outcomes when LPS was started within 48 h after oocyte retrieval versus a delayed initiation between 48 h and 96 h after oocyte retrieval. The addition of gonadotropin-releasing hormone (GnRH) agonists to progesterone vaginal pessaries showed a significant benefit in terms of live birth (OR 1.39, 95% CI 1.08 to 1.78). Only human chorionic gonadotropin (HCG) was found to be more efficacious than the placebo/no LPS treatment in terms of live birth (OR 15.43, 95% CI 2.03 to 117.12, low evidence). Any active LPSs (except for rectal or subcutaneous progesterone) was significantly more efficacious than the placebo/no LPS treatment in terms of ongoing pregnancy, with ORs ranging between 1.77 (95% CI 1.08 to 2.90) for the vaginal progesterone pessary and 2.14 (1.23 to 3.70) for the intramuscular progesterone treatment. Among the comparisons of efficacy and tolerability between the active treatments, the differences were small and very uncertain.

**Conclusion:**

Delays in progesterone supplementation until 96 h after oocyte retrieval does not affect pregnancy outcomes. The safety of GnRH agonists during the luteal phase needs to be evaluated in future studies before the applications of these agonists in clinical practice. With comparable efficacy and acceptability, there may be several viable clinical options for LPS.

**Supplementary Information:**

The online version contains supplementary material available at 10.1186/s12958-021-00782-5.

## Introduction

Assisted reproductive technology (ART) is a widely accepted procedure for couples seeking fertility aid due to infertility, with more than 1 million cycles performed every year throughout the world [1]. Supraphysiological levels of steroids that result from controlled ovarian stimulation (COS) in ART cycles are thought to inhibit pituitary luteinizing hormone secretion, thus shortening the luteal phase, which consequently causes luteal phase deficiency [2, 3]. To overcome this issue, various exogenous luteal phase supports (LPSs), including progesterone, human chorionic gonadotropin (HCG), oestrogen, gonadotropin-releasing hormone (GnRH) agonists or combinations of several of these support types, have been used to compensate for the progesterone levels; however, there is still significant debate regarding optimal LPS use.

A Cochrane review in 2015 evaluated the relative effectiveness and safety of the available methods of LPS for women undergoing ART, and the authors found no conclusive evidence regarding progesterone, HCG or the addition of oestrogen [3]. Due to the fact that some studies were not included [4–15], as well as the fact that many new randomized controlled trials (RCTs) have recently been published [16–31], the performance of a current, updated systematic review and meta-analysis at this time is warranted. Recently, several meta-analyses have examined the efficacies of oral progesterone, subcutaneous progesterone and GnRH agonists that are used during the luteal phase [32–35]. These approaches provided limited insights into the treatment hierarchy which made it difficult for clinicians to choose an optimal LPS; therefore, it is necessary to synthesize both direct and indirect available evidence from existing trials to compare the relative effects of multiple treatment options.

With regard to the timing of progesterone initiation, a systematic review recommended the initiation of LPS between the evening of oocyte retrieval and 3 days after oocyte retrieval; however, the article included limited studies that exhibited with obvious heterogeneity [36]. A recent RCT found a significantly lower ongoing pregnancy rate when starting LPS on the oocyte retrieval day, compared with day five after embryo transfer (ET) [19]. When considering the potential benefits of delaying vaginal progesterone and the shortage of meta-analyses, it would be essential to compare different progesterone initiations during the luteal phase.

This study aimed to perform a systematic review and network meta-analysis by comparing multiple LPS treatments for women with fresh cycles of in vitro fertilization (IVF) or intracytoplasmic sperm injection (ICSI), in order to inform clinical practice. Furthermore, we evaluated the effects of different initiations of LPS on the pregnancy outcomes.

## Methods

### Search strategy and selection criteria

We searched Ovid MEDLINE, the Cochrane Library, Embase, Web of Science, ClinicalTrials.gov and Google Scholar for RCTs published from the date of database inception to October 27th, 2018 and updated the search on January 9th, 2021. No language limit was applied. We used MeSH headings “fertilization in vitro”, “sperm injections, intracytoplasmic”, “ET”, “luteal phase”, “progesterone”, “chorionic gonadotropin”, “chorionic gonadotropin, beta subunit, human”, “gonadotropin-releasing hormone”, “oestrogens”, “estradiol”, and combined them with text words and word variants. The reference lists of selected articles and reviews were hand searched to identify any relevant articles. Study authors were contacted to supplement incomplete reports of the original papers. Detailed search strategy for Ovid MEDLINE can be found in Appendix S1.

### Study selection

We followed the PRISMA guidelines for network meta-analysis [37]. The study protocol was registered with PROSPERO (CRD42018115011). We selected studies for inclusion in two stages. Conference abstracts, duplicates or irrelevant articles were excluded by screening the titles and abstracts, and all of the remaining articles were screened via their full texts. Two authors independently did the screening and assessed them for eligibility with discrepancies resolved with an additional reviewer. We included RCTs that compared any agent that was used for LPS in a IVF/ICSI fresh cycle. We excluded crossover trials and quasi-RCTs. We also excluded studies involving intrauterine insemination, gamete intrafallopian transfer, zygote intrafallopian transfer and embryo transfer from donated oocytes or in vitro maturation cycles.

The participants included subfertile women undergoing IVF/ICSI fresh cycles for any reason. For inclusion, at least 10 women were required in any intervention or control group in the studies. Studies were excluded for the preservation of homogeneity if inconsistent procedures (such as different COS) were performed before oocyte retrieval in the groups. Studies including GnRH or dual triggering were also excluded. Studies had to include at least two of the following LPS categories: “any type, dose or route of progesterone which had to be provided at least five doses or continued beyond positive pregnancy test”, “any type, dose or route of HCG which had to be provided at least two doses”, “aforementioned progesterone with any type, dose or route of HCG”, “aforementioned progesterone with any type, dose or route of GnRH agonists”, “aforementioned progesterone with any type, dose or route of oestrogen”, “other interventions found” and “placebo or no LPS treatment”. Complex or rarely used LPSs were excluded.

### Study quality assessment and data extraction

Two independent reviewers undertook study quality assessment and data extraction. Any discrepancies were resolved by discussion within the review team. We assessed the risk of bias according to the Cochrane Handbook for Systematic Reviews of Interventions [38]. Specifically, attention was focused on seven domains, i.e., random sequence generation, allocation concealment, blinding of participants and personnel, blinding of outcome assessment, incomplete outcome data, selective reporting and other biases. The reviews categorized studies as “low risk”, “high risk” or “unclear risk” of bias.

Relevant information from the included trials was extracted with a predefined data extraction sheet. The extracted data included study characteristics (including published year, country and study sample size), patient characteristics (eligibility criteria), procedures (COS protocol, criteria of triggering, ovulation triggering, day of oocyte retrieval, fertilization, day of ET and ET policy), interventions (initiation, protocol and duration of LPS) and outcomes (live birth, clinical pregnancy, ongoing pregnancy, miscarriage and adverse effects).

We considered live birth and ongoing pregnancy for our primary analyses. Live birth was defined as the delivery of one or more living infants. Ongoing pregnancy was defined as a pregnancy beyond 12 weeks’ gestation. Our secondary outcomes included clinical pregnancy (defined as the presence of a gestational sac, with or without a fetal heartbeat on ultrasonography), miscarriage (defined as pregnancy loss after confirmation of clinical pregnancy) and adverse effects (including ovarian hyperstimulation syndrome [OHSS] and other system disorders). If pregnancy was not further stated, then we regarded it as being a clinical pregnancy. In cases of certain discrepancies in the definition, we accepted the primary study authors’ definition, when relevant.

### Data synthesis and statistical analysis

We categorized the interventions according to the different types, routes and combinations of LPS. When considering comparable pregnancy outcomes, we combined vaginal tablets with vaginal suppositories, and an aggregation of the different doses of progesterone was performed [39–42]. When multiple doses of progesterone were used within a trial, a pooled amount of the data was used. The odds ratios (ORs) and risk differences were calculated for all of the outcomes with 95% confidence intervals (CIs). For network meta-analysis, we used a continuity correction for studies with no events by adding 0.5 to both the events count and the total sample size.

We did two separate analyses. First, when considering comparable pregnancy rates when beginning LPS on the evening of oocyte retrieval or 1 day later, we classified the initiation of LPS as being early (within 48 h after oocyte retrieval) and delayed (48–96 h after oocyte retrieval) [18]. We performed a multivariate network meta-analysis to evaluate the effects of different initiations of LPS, and we speculated that there would be no significant differences.

Second, we analysed all of the interventions (regardless of initiation). We performed pairwise meta-analyses of the pregnancy outcomes with the random-effects model (the Mantel-Haenszel method). We assessed statistical heterogeneity in each pairwise comparison with the I^2^ statistic and *p* value [43]. To visualize the network geometry and node connectivity, we produced network plots for the outcomes [44]. Afterwards, we performed a network meta-analysis by using the methodology of the multivariate meta-analysis model. We prepared league tables presenting mixed comparisons for the inspections of both types of evidence [45]. We estimated the ranking probabilities for all of the treatments of being at each possible rank for each intervention and the treatment hierarchy was summarized as the surface under the cumulative ranking curve [46].

The assessment of statistical heterogeneity in the networks was based on the magnitude of the heterogeneity variance parameter (τ2) estimated from the network meta-analysis models. To check the assumption of consistency in the entire network, we used the design-by-treatment model and judged the presence of inconsistency based on a Chi^2^ test [47]. Local inconsistency between direct and indirect sources of evidence was statistically assessed by calculation of the difference between direct and indirect estimates in all closed loops in the network [44].

To evaluate the presence of small study effects, we visually inspected the comparison-adjusted funnel plots for the pregnancy outcomes [44]. We conducted sensitivity analyses to assess the robustness of our findings by excluding trials that were published before 2010. We prepared analyses of the data in the following subgroups (if enough studies were available): 1. different COS protocols; 2. participants with previously failed cycles, including ≤ two failed ART cycles and > two failed ART cycles; and 3. the number of embryos transferred, including single or more than one transferred embryos. The certainty of the evidence produced by the synthesis for each outcome was evaluated by using the GRADE approach and the framework described by Salanti and colleagues [46, 48]. Statistical analyses were performed with the use of STATA (version 14.0).

## Results

**Fig. 1:**
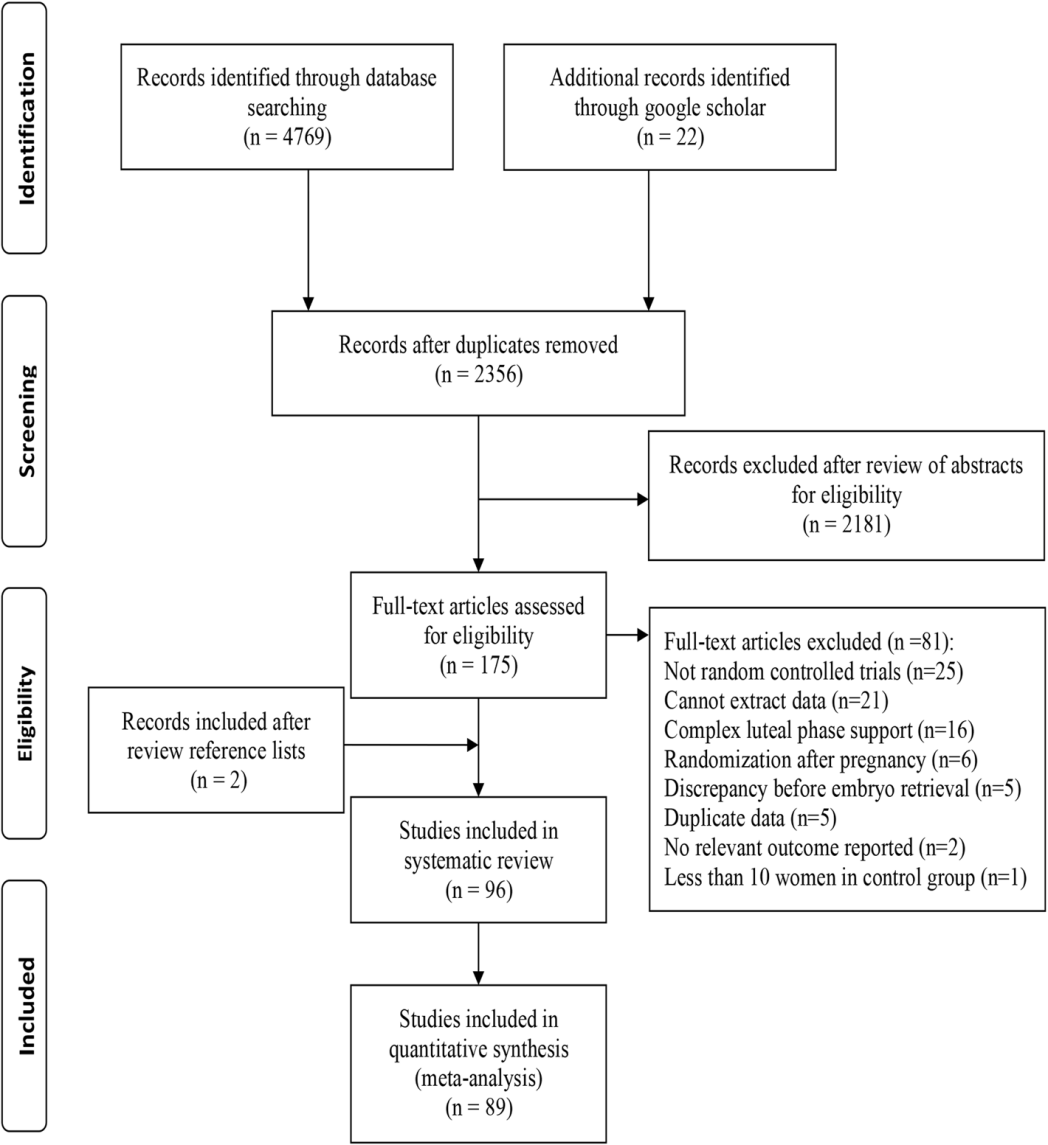
Article retrieval and screening

Overall, 4791 citations were identified with the search and 175 potentially eligible articles were retrieved in the full text. We excluded 81 reports but included two additional studies after reviewing the reference lists, thus resulting in 96 publications that were published between 1987 and 2020 (Fig. 1 and Table S1, full references for all of the trials are provided in Appendix S2). Seven studies were excluded from the meta-analysis for various reasons (Appendix S2).

Overall, 89 RCTs with 29,625 women comparing 14 interventions or placebo/no LPS treatments were included in the meta-analysis. Information concerning the risk of bias was obtained from three authors (Aghahosseini Marzieh, Bergh Christina and Nasrin Saharkhiz). Information concerning ovarian stimulation and embryo transfer was obtained from two authors (Chi HB and Monique H.Mochtar). The mean study sample size was 333 participants, ranging between 30 and 1983 patients. No study was found that involved the performance of single ET. Vaginal progesterone gels, tablets or suppositories were more commonly used than other LPSs. In terms of study quality, 52 trials were classified as exhibiting a low risk for random sequence generation. Most studies lacked the blinding of participants, personnel or outcome assessment (Figure S1 and S2).

Table 1 shows the study and patient characteristics in terms of live birth and ongoing pregnancy. The median age of the patients was 32.1 years for ongoing pregnancy. Most trials recruited women from North America, Europe and Asia. More than half of the trials used a long GnRH agonist protocol. ICSI was used in approximately 50% of trails. Seventy-two percent of the trails performed LPS within 48 h after oocyte retrieval.

**Table 1:** General characteristics of studies. Values are numbers (percentages) unless stated otherwise

Characteristics	Live birth (***n*** = 25)	Ongoing pregnancy (***n*** = 43)
**Study characteristics**		
Median (range) study sample size	355 (38–1983)	237 (38–1983)
Continent		
North America	5 (20)	9 (21)
Europe	9 (36)	17 (40)
Asia	7 (28)	11 (26)
World wide	2 (8)	2 (5)
Other	2 (8)	4 (9)
Type of interventions/controls		
Placebo/no LPS treatment	2 (8)	6 (14)
HCG	2 (8)	5 (12)
Progesterone	24 (96)	39 (91)
Combined^a^	6 (24)	11 (26)
**Patient characteristics**		
Median (range) age (years); No in group	32.4 (28.4–35.5); *n* = 24	32.1 (28.4–35.4); *n* = 40
Ovarian stimulation		
Long GnRH agonist protocol	14 (56)	24 (56)
GnRH antagonist protocol	1 (4)	5 (12)
Other protocol	4 (16)	6 (14)
Combined	6 (24)	8 (19)
Fertilization		
IVF	13 (52)	23 (53)
ICSI	2 (8)	7 (16)
IVF/ICSI	10 (40)	13 (30)
Timing LPS after oocyte retrieval		
Within 48 h	18 (72)	31 (72)
48–96 h	4 (16)	12 (28)
Not stated	5 (20)	5 (12)

^a^ Combined progesterone with HCG, oestrogen or GnRH agonists. *Abbreviations LPS* luteal phase support, *HCG* human chorionic gonadotrophin, *GnRH* gonadotropin releasing hormone, *IVF* in vitro fertilization, *ICSI* intracytoplasmic sperm injection

**Fig. 2:**
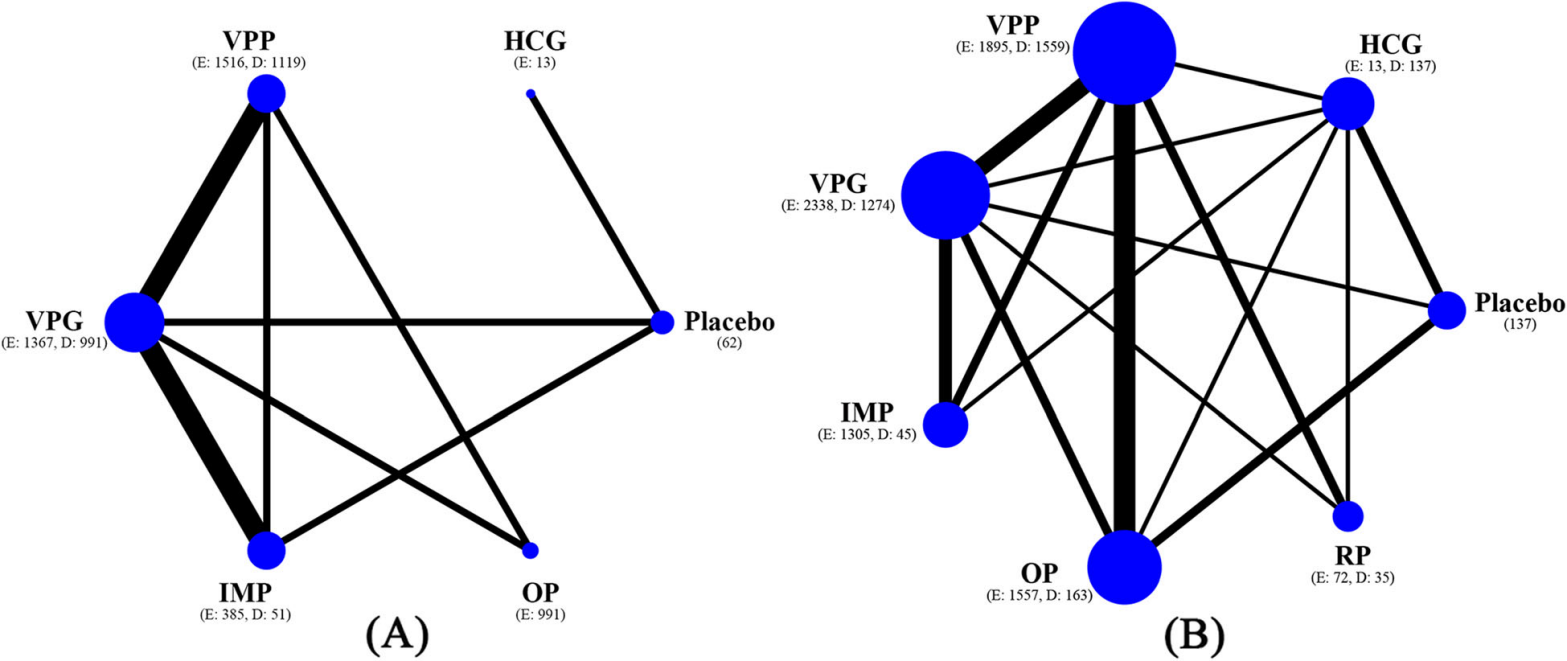
Network plots of comparisons on live birth and ongoing pregnancy of different luteal phase supports in patients undergoing fresh cycles. **A** Live birth; **B** Ongoing pregnancy. Size of node is proportional to number of arms randomized to each treatment (numbers of subgroup patients with early and delayed luteal phase supports are presented in brackets). Line width is proportional to number of randomized controlled trials comparing each pair of treatments. HCG = human chorionic gonadotrophin; VPP = vaginal progesterone pessary; VPG = vaginal progesterone gel; IMP = intramuscular progesterone; OP = oral progesterone; RP = rectal progesterone

**Fig. 3:**
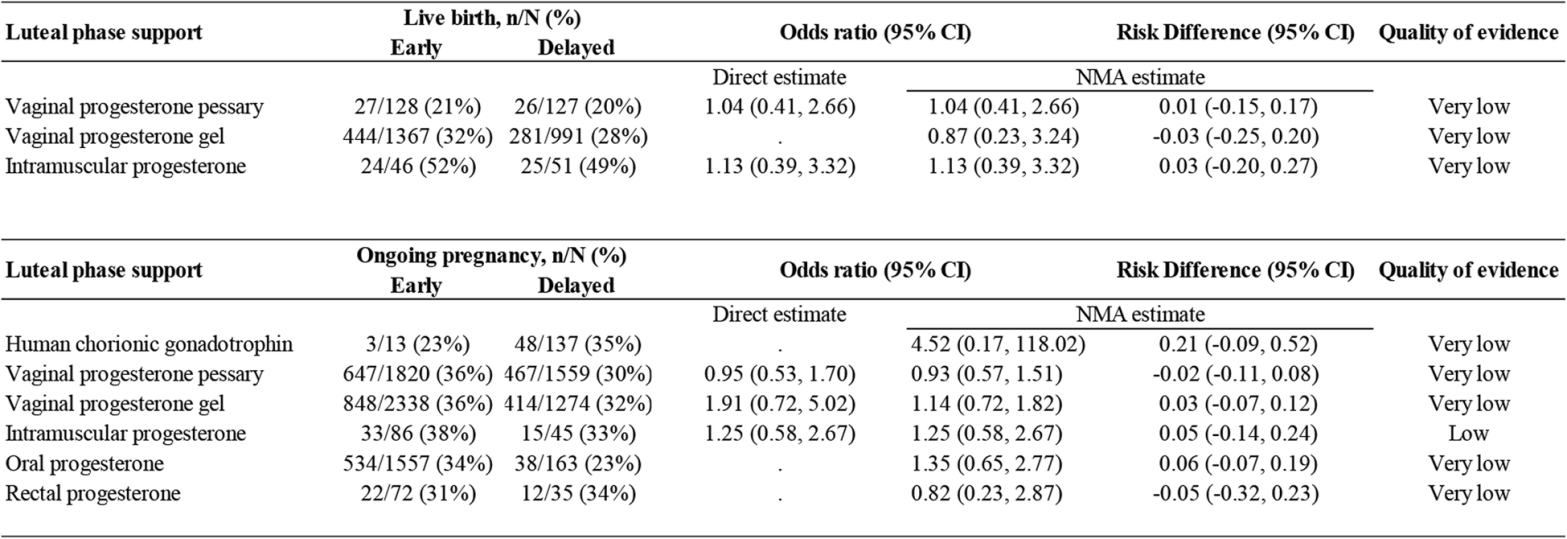
Network meta-analysis (NMA) of live birth and ongoing pregnancy according to luteal phase support initiation strategy. Abbreviations: HCG = human chorionic gonadotrophin; VPP = vaginal progesterone pessary; VPG = vaginal progesterone gel; IMP = intramuscular progesterone; OP = oral progesterone; RP = rectal progesterone

Figure 2 shows the network of eligible comparisons of LPS in different initiations for the primary outcomes. The results of the network meta-analysis for pregnancy outcomes are presented in Fig. 3 and Figure S3. The early administration of progesterone showed no evidence of an effect on live birth being greater than the delayed (ORs ranging from 0.87 for vaginal progesterone gel to 1.14 for intramuscular progesterone, with a very low degree of evidence). When compared with delayed LPS, early LPS was not associated with significantly higher ongoing pregnancy rates, with estimated ORs ranging from 0.82 (95% CI 0.23 to 2.87) for rectal progesterone to 4.52 (95% CI 0.17 to 118.02) for HCG. The evidence was either low or very low for these results. No significant differences were found in any of the comparisons in terms of clinical pregnancy and miscarriage. A fitting of the design-by-treatment interaction model provided no evidence for statistically significant inconsistency for pregnancy outcomes (*P* = 0.11 to 0.68). We found no significant evidence for local inconsistency in all of the closed loops (Figure S4).

**Fig. 4:**
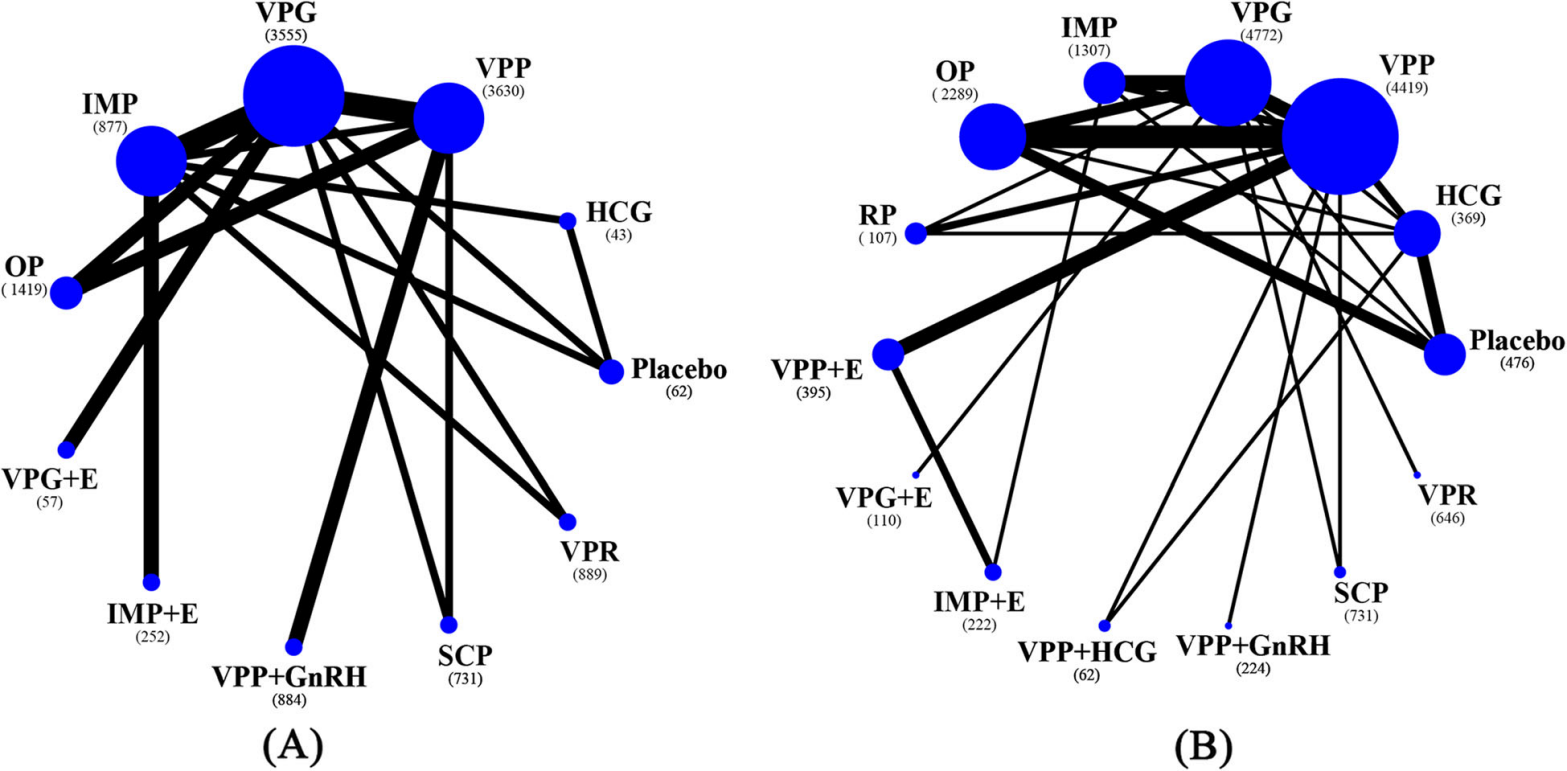
Network plots of comparisons on live birth and ongoing pregnancy (regardless of the initiations of luteal phase supports). **A** Live birth; **B** Ongoing pregnancy. Size of node is proportional to number of arms randomized to each treatment (number of patients in brackets). Line width is proportional to number of randomized controlled trials comparing each pair of treatments. HCG = human chorionic gonadotrophin; VPP = vaginal progesterone pessary; VPG = vaginal progesterone gel; IMP = intramuscular progesterone; OP = oral progesterone; RP = rectal progesterone; GnRH = gonadotropin releasing hormone; E = oestrogen; SCP = subcutaneous progesterone; VPR = vaginal progesterone ring

**Fig. 5:**
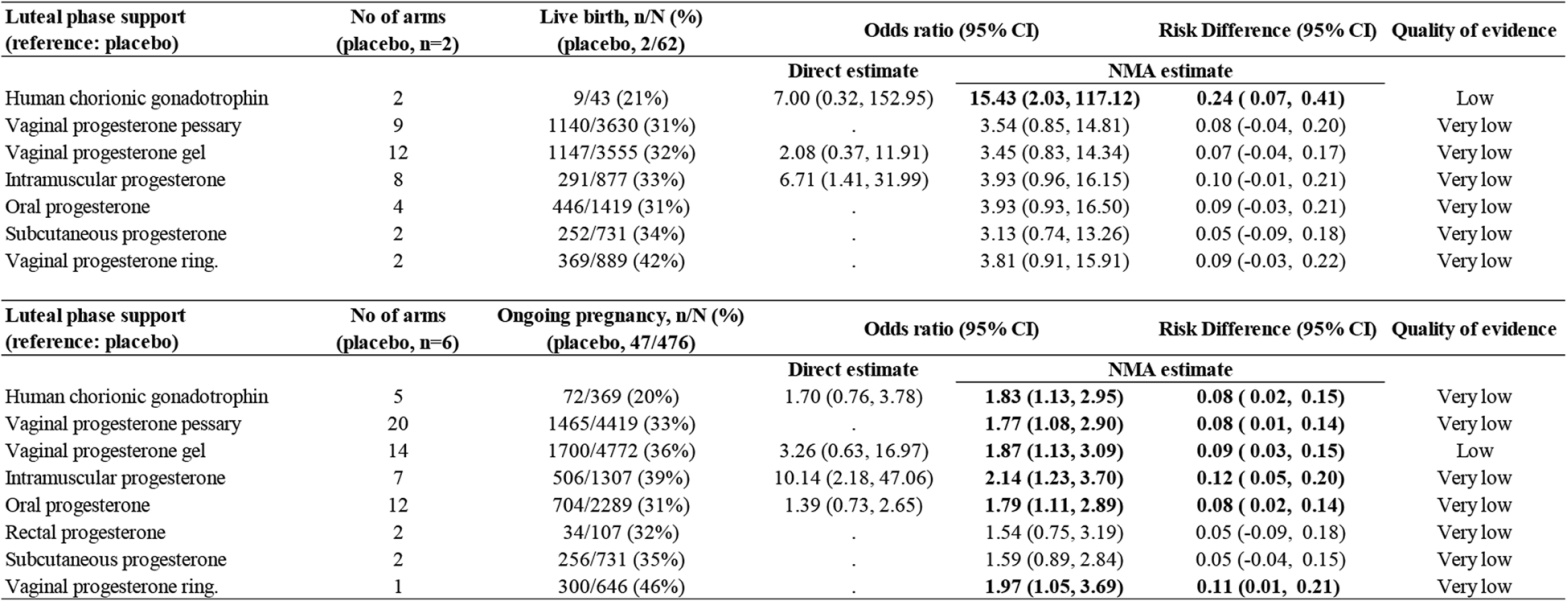
Network meta-analysis (NMA) for live birth and ongoing pregnancy

**Fig. 6:**
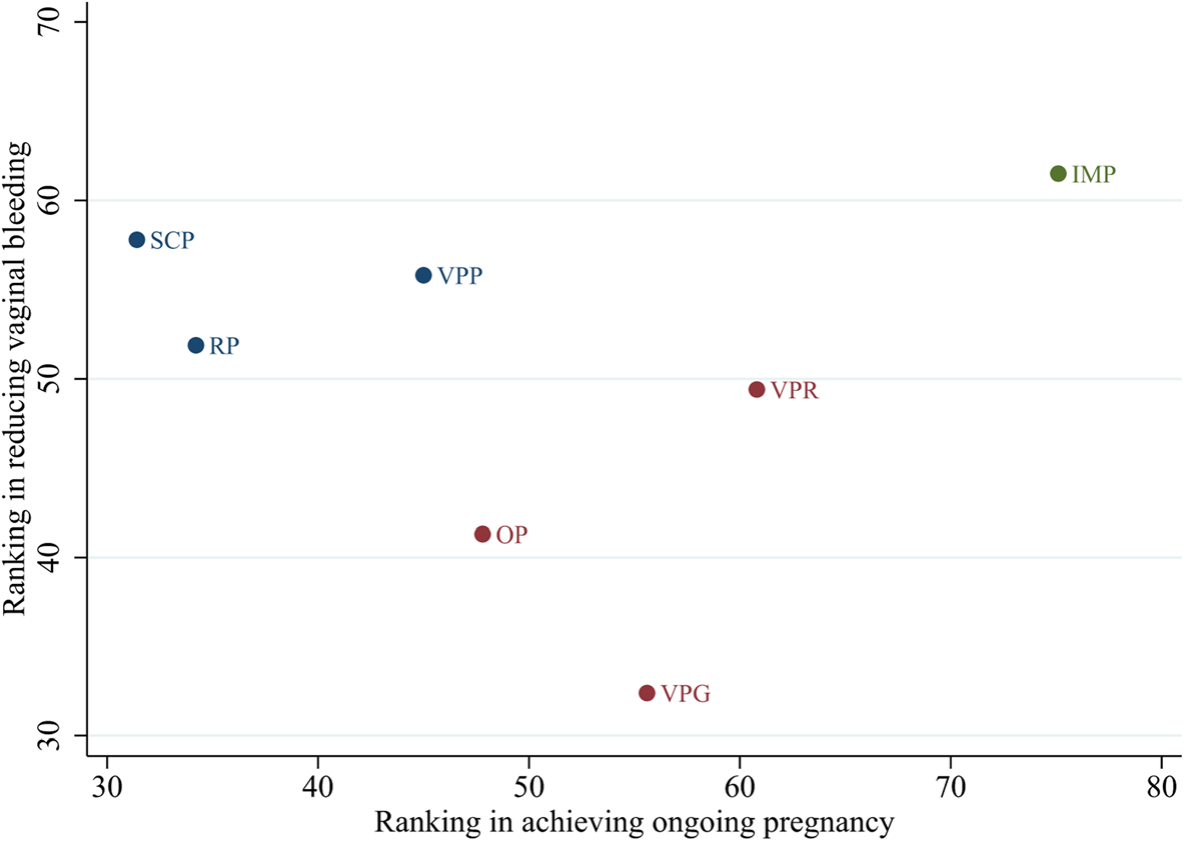
Clustered ranking plot by mean rank values from results of network meta-analyses of ongoing pregnancy and vaginal bleeding. VPP = vaginal progesterone pessary; VPG = vaginal progesterone gel; IMP = intramuscular progesterone; OP = oral progesterone; RP = rectal progesterone; SCP = subcutaneous progesterone; VPR = vaginal progesterone ring

Figure 4 shows the network of eligible comparisons of LPS for primary outcomes. Only HCG was found to be more efficacious than placebo/no LPS treatment in terms of live birth (OR 15.43, 95% CI 2.03 to 117.12, low evidence, Fig. 5). Placebo treatments were significantly less efficacious than any active LPS (except for rectal or subcutaneous progesterone) in terms of ongoing pregnancy, and the ORs for LPS associated with significant improvements ranged between 1.77 (95% CI 1.08 to 2.90) for vaginal progesterone pessary and 2.14 (95% CI 1.23 to 3.70) for intramuscular progesterone (Fig. 5). Progesterone that was applied vaginally, orally or intramuscularly was found to be associated with a higher chance of clinical pregnancy (Figure S5). With regard to the effects of adding oestrogen, HCG or GnRH agonists in the luteal phase, the additions of oestrogen or GnRH agonists to the vaginal progesterone pessary showed a significant benefit in terms of some of the pregnancy outcomes (Figure S6). In terms of tolerability, vaginal bleeding was counted in more studies than other adverse events. There was no strong evidence of any significant difference when comparing vaginal progesterone pessary with other types (Figure S7). A network meta-analysis of OHSS was not performed, due to the absence of a closed loop. The mean rank of each treatment was plotted to illustrate clustering of interventions according to higher effectiveness (achieving ongoing pregnancy) and higher acceptability (reducing vaginal bleeding). Although intramuscular progesterone ranked higher on both outcomes, the probability of other adverse events was not reported in previous studies (Fig. 6).

The test of global inconsistency showed no significant difference between the consistency and inconsistency models for pregnancy outcomes (*P* = 0.08 to 0.82). We found no significant evidence for local inconsistency in all of the closed loops (Figure S8). Additionally, we found no strong evidence of small study effects across outcomes in the network meta-analysis (Figure S9). Predefined subgroup analyses were not available, due to the limited number of studies in some of the subgroups. Therefore, we performed a sensitivity analysis by including articles that used the long GnRH agonist protocol. The sensitivity analyses did not affect the main results (Figure S10).

## Discussion

This analysis was based on 89 trials, which included 29,625 couples with infertility randomly assigned to active LPS or placebo/no LPS treatments. To our knowledge, this study represents the most comprehensive synthesis of data for currently available LPSs for women undergoing IVF/ICSI. No significant differences were found in terms of all of the pregnancy outcomes when LPS was started within 48 h after oocyte retrieval versus delayed initiation between 48 h and 96 h after oocyte retrieval. The additions of oestrogen or HCG to regular LPS did not improve pregnancy outcomes in most cases. The administration of a GnRH agonist during the luteal phase in women undergoing IVF/ICSI was found to improve live birth and clinical pregnancy. The placebo was significantly less efficacious than any active LPS (except for rectal or subcutaneous progesterone) in terms of ongoing pregnancy and clinical pregnancy. Of the active comparisons for pregnancy and adverse outcomes, the precision of the summary treatment effect estimates varied considerably, with higher levels of uncertainty for treatments for which there were only a few available RCTs.

This was the first meta-analysis performed to compare the effects of different initiations of LPS on pregnancy outcomes, and the results were consistent with those of a previous systematic review [36]. This article did not evaluate live birth and ongoing pregnancy, which were the primary outcomes in our work. Feichtinger et al. conducted a RCT including 910 women undergoing ET 3 days after oocyte retrieval, and the group that initiated LPS 1 day after oocyte retrieval did not show any significant differences concerning pregnancy rates, compared with the group that started LPS 4 days after oocyte retrieval [49]. In this RCT, the LPS consisted of a combination of vaginal progesterone pessary, oral progesterone and oestrogen. In our study, we evaluated the different application routes of luteal phase support, which provided more evidence for the use of delayed LPS. Ghanem ME and colleagues found that the initiation of LPS on the oocyte retrieval day was associated with a poorer cycle outcome on day five (but not on day three) ET, when compared with the initiation of LPS on the ET day [19]. Williams et al. reported of a lower pregnancy rate in patients who started progesterone on day six after oocyte retrieval, compared with day three after oocyte retrieval; however, this article performed ET 3 days after oocyte retrieval [50]. In our review, only these two studies performed LPS until 96 h after oocyte retrieval, and we could not evaluate the influence of further delaying LPS until later timepoints [19, 50]. Future studies are needed to address the gaps in the evidence.

Consistent with most of our results, a previous meta-analysis found no benefit of the addition of oestrogen including transdermal administrations during the luteal phase for improving IVF/ICSI outcomes [51]. Due to the limited studies and the very low evidence, the potential benefit of oestrogen that was found in our results should be interpreted with caution and needs to be evaluated in further studies. Although our results support the benefit of the addition of GnRH agonists during the luteal phase, which has been reported in several previous meta-analyses [3, 35, 52], such a benefit, if present, is unlikely to promote the application of GnRH agonists in clinical practice until their safety (including both adverse perinatal outcomes and congenital malformations) can be confirmed in future studies.

HCG seems to yield improvements in live birth rates; however, the results have mainly been based on older and small-scale studies. Concerning its higher risk of OHSS, HCG was not used in trials published after 2010 [3]. Rectal or subcutaneous progesterone seem to be less efficacious than other active LPSs; however, no significant differences were found, and the result was consistent with that of a previous analysis [33]. When considering the comparable pregnancy rates among other LPS types, we found limited evidence to recommend any LPS as being the first-line protocol for IVF/ICSI cycles. We also found a comparable miscarriage rate between the placebo and active LPS treatments, which was due to the lower clinical pregnancy rate in the control groups using the placebo. Our findings for tolerability were mainly based on limited studies, and no definite conclusion could be made at the current stage.

There were several important limitations in this study. Many of the included studies exhibited an unclear risk of bias, and most of the comparisons were assessed as being low or very low in quality, which largely restricts the interpretation of these results. Moreover, most of the studies lacked the blinding of participants, personnel or outcome assessments; nevertheless, it is unlikely that pregnancy outcomes (such as live birth or miscarriage) can be affected by a detection bias. Publication bias is always a concern, and we searched ClinicalTrials.gov and included non-English language trials. Additional information was obtained from the original authors. Some adverse events were reported in the limited number of studies, and these events were not compared in our analysis. Similarly, a cost-effectiveness analysis could not be performed in our work, which is a major concern for clinical practice.

The findings of this comprehensive network meta-analysis provide some evidence to support the late initiation of LPS, thereby reducing both costs and side effects. Herein, we provide the best available evidence suggesting that the additions of oestrogen or HCG to progesterone for LPS via different administration routes or doses do not improve the pregnancy rate. Additionally, we provide the treatment hierarchy, as well as the ORs, for pregnancy outcomes and adverse events among active uses of LPS, which can enable the personalization of clinically effective treatments without a major risk of adverse effects for women undergoing fresh IVF/ICSI cycles. The safety of adding GnRHs agonist during the luteal phase was not well studied in this population, and further research should be conducted to accumulate more evidence concerning this effect.

## Conclusion

Delaying progesterone supplementation until 96 h after oocyte retrieval does not affect pregnancy outcomes. The current evidence does not support the addition of oestrogen or HCG during the luteal phase. The safety of GnRH agonists during the luteal phase needs to be evaluated in future studies before their application in clinical practice. There were no significant differences in the efficacy and acceptability profiles across the treatments, and the best choice may not be uniform across patients.

## Supplementary Information

**Additional file 1: Appendix S1.** MEDLINE search strategy. **Appendix S2.** Full references for all trials included in the review.

**Additional file 2: Figure S1.** Risk of bias presented as percentages across all included studies. **Figure S2.** Summary of risk of bias for each trial. **Figure S3.** Network meta-analysis (NMA) of clinical pregnancy and miscarriage according to luteal phase support initiation strategy. Abbreviations: HCG=human chorionic gonadotrophin; VPP=vaginal progesterone pessary; VPG=vaginal progesterone gel; IMP=intramuscular progesterone; OP=oral progesterone; RP=rectal progesterone. **Figure S4.** Global and local inconsistency tests between direct and indirect estimates in the analyses of the pregnancy outcomes. Blue alphabets represent early luteal phase supports. Yellow alphabets represent delayed luteal phase supports. A=placebo; B=human chorionic gonadotrophin; C=vaginal progesterone pessary; D=vaginal progesterone gel; E=intramuscular progesterone; F=oral progesterone; G=rectal progesterone. **Figure S5.** Network meta-analysis (NMA) for clinical pregnancy and miscarriage. **Figure S6.** The effects of adding oestrogen, HCG or GnRH agonists in the luteal phase on the pregnancy outcomes. Abbreviations: GnRH, gonadotropin releasing hormone; HCG, human chorionic gonadotrophin. **Figure S7.** The effects of luteal phase support on adverse events. **Figure S8.** Global and local inconsistency tests between direct and indirect estimates in the analyses of the pregnancy outcomes (regardless of the initiations of luteal phase supports). A=placebo; B=human chorionic gonadotrophin; C=vaginal progesterone pessary; D=vaginal progesterone gel; E=intramuscular progesterone; F=oral progesterone; G=rectal progesterone; H=vaginal progesterone pessary+ oestrogen; J=intramuscular progesterone+ oestrogen; K= vaginal progesterone pessary + human chorionic gonadotrophin; L=intramuscular progesterone+ human chorionic gonadotrophin; N=subcutaneous progesterone; O=vaginal progesterone ring. **Figure S9.** Comparison-adjusted funnel plot for the pregnancy outcomes. **Figure S10.** Network meta-analysis (NMA) for ongoing pregnancy. (A) including studies since 2010, (B) including articles using long gonadotropin releasing hormone agonist protocol.

**Additional file 3: Table S1.** Characteristics of the included studies in the systematic review. Abbreviations: NS, not stated; IVF, in vitro fertilization; ICSI, intracytoplasmic sperm injection; GnRH, gonadotropin releasing hormone; HCG, human chorionic gonadotrophin; FSH, follicle stimulating hormone; LH, luteinizing hormone; hMG, human menopausal gonadotropin; ET, embryo transfer; PD, pituitary desensitisation; SC, subcutaneous; IM, intramuscular; ART, assisted reproductive technology; BMI, body mass index; OHSS, ovarian hyperstimulation syndrome.

## Data Availability

The current study was based on the results of relevant published studies.

## Abbreviations

ART: Assisted reproductive technology
COS: Controlled ovarian stimulation
LPSs: Luteal phase supports
HCG: Human chorionic gonadotropin
GnRH: Gonadotropin-releasing hormone
RCTs: Randomized controlled trials
ET: Embryo transfer
IVF: In vitro fertilization
ICSI: Intracytoplasmic sperm injection
OHSS: Ovarian hyperstimulation syndrome
ORs: Odds ratios
CIs: Confidence intervals
