# Supplementary material for: Luteal phase support for in vitro fertilization/intracytoplasmic sperm injection fresh cycles: a systematic review and network meta-analysis
Source: Reprod Biol Endocrinol. 2021 Jul 6;19:103. doi: 10.1186/s12958-021-00782-5 (PMC8259396; doi:10.1186/s12958-021-00782-5)
Supplement: Supplementary file 1 — Additional file 1: Appendix S1. MEDLINE search strategy. Appendix S2. Full references for all trials included in the review. [file 12958_2021_782_MOESM1_ESM.pdf]

Appendix S1. MEDLINE search strategy (from inception until Jan 9th, 2021)

1 exp sperm injections, intracytoplasmic/ or exp fertilization in vitro/ or exp embryo transfer/

2 embryo transfer$.tw.

3 in vitro fertilization.tw.

4 ivf - et.tw.

5 (ivf or et).tw.

6 icsi.tw.

7 intracytoplasmic sperm injection$.tw.

8 (blastocyst adj2 transfer$).tw.

9 embryo transfer$.tw.

10 or/1 - 9

11 exp luteal phase/

12 (luteal adj5 support$).tw.

13 (luteal adj5 phase).tw.

14 (ischemic adj5 phase).tw.

15 post ovulat$.tw.

16 (post adj5 transfer$).tw.

17 (after adj5 transfer$).tw.

18 (post adj5 trigger$).tw.

19 (after adj5 trigger$).tw.

20 or/11 - 19

21 10 and 20

22 exp progesterone/

23 progesterone$.tw.

24 dydrogesterone.tw.

25 utrogest.tw.

26 17 alpha - hydroxyprogesterone.tw.

27 prontogest.tw.

28 exp chorionic gonadotropin/ or exp chorionic gonadotropin, beta subunit, human/

29 HCG.tw.

30 crinone.tw.

31 chorionic gonadotropin$.tw.

32 chorionic gonadotrophin$.tw.

33 exp gonadotropin - releasing hormone/

34 gnrha.tw.

35 gnrh agonist$.tw.

36 gnrh a.tw.

37 gonadotropin - releasing hormone agonist$.tw.

38 exp buserelin/ or exp goserelin/ or exp leuprolide/ or exp nafarelin/ or exp triptorelin pamoate/

39 leuprolide.tw.

40 triptorelin.tw.

41 goserelin.tw.

42 nafarelin.tw.

43 buserelin.tw.

44 exp estrogens / or exp estradiol

45 estrogen$.tw.

46 estradiol.tw.

47 oestradiol.tw.

48 progynova.tw.

49 vivelle.tw.

50 aerodiol.tw.

51 estrace.tw.

52 estraderm.tw.

53 delestrogen.tw.

54 ovocyclin.tw.

55 or/22 - 54

56 21 and 55

57 randomized controlled trial.pt.

58 controlled clinical trial.pt.

59 randomized.ab.

60 randomised.ab.

61 placebo.tw.

62 clinical trials as topic.sh.

63 randomly.ab.

64 trial.ti.

65 or/57 - 64

66 exp animals/ not humans.sh.

67 65 not 66

68 56 and 67

Appendix S2. Full references for all trials included in the review (with reasons excluding from meta-analyses)

1. Abate A, Brigandi A, Abate FG, Manti F, Unfer V, Perino M. Luteal phase support with 17α-hydroxyprogesterone versus unsupported cycles in in vitro fertilization: A comparative randomized study. Gynecol Obstet Invest. 1999;48:78-80.
2. Abate A, Perino M, Abate FG, Brigandì A, Costabile L, Manti F. Intramuscular versus vaginal administration of progesterone for luteal phase support after in vitro fertilization and embryo transfer. A comparative randomized study. Clin Exp Obstet Gynecol. 1999;26:203-6.
3. Aboulghar MA, Marie H, Amin YM, Aboulghar MM, Nasr A, Serour, GI, Mansour RT. GnRH agonist plus vaginal progesterone for luteal phase support in ICSI cycles: a randomized study. Reprod Biomed Online. 2015;30:52-6.
4. Aflatoonian A, Asgharnia M, Seyed Alshohadaei F. Comparison of progesterone administration before and after embryo transfer in ART cycles. J Reprod Infertil. 2004;5:44-51.
5. Aghahosseini M, Aleyassin A, Khodaverdi S, Esfahani F, Mohammadbeigi R, Movahedi S, Kord VA, Mahdavi A, Fallahi P, Shabani P, et al. Estradiol supplementation during the luteal phase in poor responder patients undergoing in vitro fertilization: a randomized clinical trial. J Assist Reprod Genet. 2011;28:785-90.
6. Aghsa MM, Rahmanpour H, Bagheri M, Davari-Tanha F, Nasr R. A randomized comparison of the efficacy, side effects and patient convenience between vaginal and rectal administration of Cyclogest when used for luteal phase support in ICSI treatment. Arch Gynecol Obstet. 2012;286:1049-54.
7. Artini PG, Volpe A, Angioni S, Galassi MC, Battaglia C, Genazzani AR. A comparative, randomized study of three different progesterone support of the luteal phase following IVF/ET program. J Endocrinol Invest. 1995;18:51-6.
8. Ashrafi M, Madani T, Sh Tehrani E, Moeini A. Vaginal progestrone versus intramuscular in assisted reproduction (a comparative study). Medical Journal of Reproduction and Infertility. 2000;1:35-9.
9. Ata B, Kucuk M, Seyhan A, Urman B. Effect of high dose estrogen in luteal phase support on live birth rates after assisted reproduction treatment cycles. J Reprod Med.2010;55:485-90.
10. Baker VL, Jones CA, Doody K, Foulk R, Yee B, Adamson GD, Cometti B, DeVane G, Hubert

G, Trevisan S, et al. A randomized, controlled trial comparing the efficacy and safety of aqueous subcutaneous progesterone with vaginal progesterone for luteal phase support of in vitro fertilization. Hum Reprod.2014;29:12-20.

11. Baruffi RLR, Franco Jr JG, Mauri AL, Petersen CG, Felipe V, Garbellini E. Effects of vaginal progesterone administration starting on the day of oocyte retrieval on pregnancy rates. Jornal brasileiro de reproducao assistida.2002;6:61-3
12. Beckers NGM, Laven JSE, Eijkemans MJC, Fauser B. Follicular and luteal phase characteristics following early cessation of gonadotrophin-releasing hormone agonist during ovarian stimulation for in-vitro fertilization. Hum Reprod.2000;15:43-9.
13. Belaisch-Allart J, Testart J, Fries N, Forman RG, Frydman R. The effect of dydrogesterone supplementation in an IVF programme. Hum Reprod.1987;2:183-5.
14. Belaisch-Allart J, De Mouzon J, Lapousterle C, Mayer M. The effect of HCG supplementation after combined GnRH agonist/HMG treatment in an IVF programme. Hum Reprod.990;5:163-6.
15. Beltsos AN, Sanchez MD, Doody KJ, Bush MR, Domar AD, Collins MG. Patients' administration preferences: progesterone vaginal insert (Endometrin®) compared to intramuscular progesterone for Luteal phase support. Reprod Health. 2014;11:78.
16. Bergh C, Lindenberg S. A prospective randomized multicentre study comparing vaginal progesterone gel and vaginal micronized progesterone tablets for luteal support after in vitro fertilization/intracytoplasmic sperm injection. Hum Reprod.2012;27:3467-73.
17. Ceyhan ST, BasaranM, Kemal Duru N, Yilmaz A, Göktolga U, Baser I. Use of luteal estrogen supplementation in normal responder patients treated with fixed multidose GnRH antagonist: a prospective randomized controlled study. Fertil Steril.2008;89:1827-30.
18. Chakravarty BN, Shirazee HH, Dam P, Goswami, Sourendra K, Chatterjee R, Ghosh S. Oral dydrogesterone versus intravaginal micronised progesterone as luteal phase support in assisted reproductive technology (ART) cycles: results of a randomised study. J Steroid Biochem Mol Biol.2005;97:416-20.
19. Chavez FCP, Delgadillo JCB, Rueda SSO, Villa GB, Acosta SV, Solis VS, Caballero MO, Gavino FG. Estrogen role in the luteal phase support in in vitro fertilization with embryo transfer cycles. Ginecol Obstet Mex.2004;72:645-55.

20. Chi H, Li R, Qiao J, Chen X, Wang X, Hao G, Wu Q, Cao Y, Cai L, Ye H, et al. Vaginal progesterone gel is non-inferior to intramuscular progesterone in efficacy with acceptable tolerability for luteal phase support: A prospective, randomized, multicenter study in China. Eur J Obstet Gynecol Reprod Biol. 2019;237:100-5.
21. Colwell KA, Tummon IS. Elevation of serum progesterone with oral micronized progesterone after in vitro fertilization. J Reprod Med. 1991;36:170-2.
22. Dal Prato L, Bianchi L, Cattoli M, Tarozzi N, Flamigni C, Borini A. Vaginal gel versus intramuscular progesterone for luteal phase supplementation: a prospective randomized trial. Reprod Biomed Online. 2008;16:361-7.
23. Doody KJ, Schnell VL, Foulk RA, Miller CE, Kolb BA, Blake EJ, Yankov VI. Endometrin for luteal phase support in a randomized, controlled, open-label, prospective in-vitro fertilization trial using a combination of Menopur and Bravelle for controlled ovarian hyperstimulation. Fertil Steril. 2009;91:1012-7.
24. Elgindy EA, El-Haieg DO, Mostafa MI, et al. Does luteal estradiol supplementation have a role in long agonist cycles? Fertil Steril 2010;93:2182-8. doi: 10.1016/j.fertnstert.2009.01.066
25. Elgindy EA, El-Haieg DO, Mostafa MI, Shafiek M. The effect of luteal phase vaginal estradiol supplementation on the success of in vitro fertilization treatment: a prospective randomized study. Fertil Steril. 2008;89:554-61.
26. Fanchin R, Righini C, de Ziegler D, Olivennes F, Ledee N, Frydman R. Effects of vaginal progesterone administration on uterine contractility at the time of embryo transfer. Fertil Steril. 2001;75:1136-40.
27. Fatemi HM, Kolibianakis EM, Camus M, Tournaye H, Donoso P, Papanikolaou E, Devroey P. Addition of estradiol to progesterone for luteal supplementation in patients stimulated with GnRH antagonist/rFSH for IVF: a randomized controlled trial. Hum Reprod. 2006;21:2628-32.
28. Friedler S, Raziel A, Schachter M, Strassburger D, Bukovsky I, Ron-El R. Luteal support with micronized progesterone following in-vitro fertilization using a downregulation protocol with gonadotrophin-releasing hormone agonist: a comparative study between vaginal and oral administration. Hum Reprod. 1999;14:1944-8.
29. Fujimoto A, Osuga Y, Fujiwara T, Yano T, Tsutsumi O, Momoeda M, Kugu K, Koga K, Morita Y, Wada O, et al. Human chorionic gonadotropin combined with progesterone for luteal support

improves pregnancy rate in patients with low late-midluteal estradiol levels in IVF cycles. J Assist Reprod Genet. 2002;19:550-4.

30. Fusi FM, Brigante CM, Zanga L, Mignini Renzini M, Bosisio C, Fadini R. GnRH agonists to sustain the luteal phase in antagonist IVF cycles: a randomized prospective trial. Reprod Biol Endocrinol. 2019;17:103.
31. Ganesh A, Chakravorty N, Mukherjee R, Goswami S, Chaudhury K, Chakravarty B. Comparison of oral dydrogesterone with progesterone gel and micronized progesterone for luteal support in 1,373 women undergoing in vitro fertilization: a randomized clinical study. Fertil Steril. 2011;95:1961-5.
32. Geber S, Moreira ACF, de Paula SOC, Sampaio M. Comparison between two forms of vaginally administered progesterone for luteal phase support in assisted reproduction cycles. Reprod Biomed Online. 2007; 14:155-8.
33. Ghanem ME, Bedairy MH, Shaaban A, Albahlol IA. Does the Time of Starting Progesterone Luteal Support Affect Embryo Transfer in Long Agonist Protocol Downregulated ICSI Cycles? A Randomized Controlled Trial. Reprod Sci. 2020; doi: 10.1007/s43032-020-00309-0.
34. Golan A, Herman A, Soffer Y, Bukovsky I, Caspi E, Ron-El R. Human chorionic gonadotrophin is a better luteal support than progesterone in ultrashort gonadotrophin-releasing hormone agonist/menotrophin in-vitro fertilization cycles. Hum Reprod. 1993;8: 1372-5.
35. Gorkemli H, Ak D, Akyurek C, Aktan M, Duman S. Comparison of pregnancy outcomes of progesterone or progesterone plus estradiol for luteal phase support in ICSI-ET cycles. Gynecol Obstet Invest. 2004;58:140-4.
36. Goudge CS, Nagel TC, Damario MA. Duration of progesterone-in-oil support after in vitro fertilization and embryo transfer: a randomized, controlled trial. Fertil Steril. 2010;94:946-51.
37. Griesinger G, Blockeel C, T Sukhikh G, Patki A, Dhorepatil B, Yang DZ, Chen ZJ, Kahler E, Pexman-Fieth C, Tournaye H. Oral dydrogesterone versus intravaginal micronized progesterone gel for luteal phase support in IVF: a randomized clinical trial. Hum Reprod. 2018;10:1-10.
38. Ibrahem MA. Oral Dydrogesterone versus Vaginal Micronized Progesterone in Luteal Phase Support after Controlled Ovarian Stimulation Using Long Gonadotropin-Releasing Hormone Agonist in Women Undergoing in Vitro Fertilization/Intracytoplasmic Sperm Injection. Open

Journal of Obstetrics and Gynecology. 2019; 09:1558-68.

39. Ismail Madkour WA, Noah B, Abdel Hamid AMS, Zaheer H, Al-Bahr A, Shaeer M, Moawad A. Luteal phase support with estradiol and progesterone versus progesterone alone in GnRH antagonist ICSI cycles: a randomized controlled study. Human Fertility. 2016;19:142-9.
40. Jiang CL, Hu DL, Peng HY, Wang H, Zhang CJ. Effects of Human Chorionic Gonadotropin Combined Progesterone as Luteal Support on the Outcome of IVF-ET in Patients with Low OHSS Risk. Journal of yunyang medical college. 2009;28:357-62.
41. Kably Ambe A, Ruiz Anguas J, Walters Arballo F, García Benitez CQ, Karchmer KS. Results' analysis of estradiol and progesterone supplementation in luteal phase vs progesterone alone in an assisted reproduction program. Ginecol Obstet Mex. 2005;73:173–82.
42. Kapur A, Prasad S, Kumar A. Is luteal phase estradiol supplementation beneficial in long agonist IVF-ET cycles? First prospective randomised controlled study from indian subcontinent. J Clin Diagn Res. 2018;12:QC01-QC03.
43. Khrouf M, Slimani S, Khrouf MR, Braham M, Bouyahia M, Berjeb KK, Chaabane HE, Merdassi G, Kaffel AZ, Zhioua A, et al. Progesterone for Luteal Phase Support in In Vitro Fertilization: Comparison of Vaginal and Rectal Pessaries to Vaginal Capsules: A Randomized Controlled Study. Clin Med Insights Womens Health. 2016;9:43-7.
44. Kleinstein J. Efficacy and tolerability of vaginal progesterone capsules (Utrogest 200) compared with progesterone gel (Crinone 8%) for luteal phase support during assisted reproduction. Fertil Steril. 2005;83:1641-9.
45. Kupferminc MJ, Lessing JB, Amit A, Yovel I, David MP, Peyser MR. A prospective randomized trial of human chorionic gonadotrophin or dydrogesterone support following in-vitro fertilization and embryo transfer. Hum Reprod. 1990;5:271-3.
46. Kutlusoy F, Guler I, Erdem M, Erdem A, Bozkurt N, Biberoglu EH, Biberoglu KO. Luteal phase support with estrogen in addition to progesterone increases pregnancy rates in in vitro fertilization cycles with poor response to gonadotropins. Gynecol Endocrinol. 2014;30:363-6.
47. Kwon SK, Kim CH, Lee KH, Jeon IK, Ahn JW, Kim SH, Chae HD, Kang BM. Luteal estradiol supplementation in gonadotropin-releasing hormone antagonist cycles for infertile patients in vitro fertilization. Clin Exp Reprod Med. 2013; 40:131-4.
48. L Cem D, Belgin S, Tolga E, Arda L, Deniz GK, Harika BÖ. Luteal Faz Desteği Başlama

Zamanlarının Randomize Karşılaştırılması. Acıbadem Üniversitesi Sağlık Bilimleri Dergisi. 2010;1:185-7.

49. Lewin A, Benshushan A, Mezker E, Yanai N, Schenker JG, Goshen R. The role of estrogen support during the luteal phase of in vitro fertilization-embryo transplant cycles: a comparative study between progesterone alone and estrogen and progesterone support. Fertil Steril. 1994;62: 121-5.
50. Licciardi F, Kwiatkowski A, Noyes N, Berkeley AS, Krey LL, Grifo JA. Oral versus intramuscular progesterone for in vitro fertilization: a prospective randomized study. Fertil Steril. 1999;71:614-8.
51. Lin H, Li Y, Li L, Wang W, Zhang Q, Chen X, Yang D. Oral oestradiol supplementation as luteal support in IVF/ICSI cycles: a prospective, randomized controlled study. Eur J Obstet Gynecol Reprod Biol. 2013;167:171-5.
52. Lockwood G, Griesinger G, Cometti B. Subcutaneous progesterone versus vaginal progesterone gel for luteal phase support in in vitro fertilization: a noninferiority randomized controlled study. Fertil Steril. 2014;101:112-9.
53. Ludwig M, Finas A, Katalinic A, Strik D, Kowalcek I, Schwartz P, Felberbaum R, Küpker W, Schöpper B, Al-Hasani S, et al. Prospective, randomized study to evaluate the success rates using hCG, vaginal progesterone or a combination of both for luteal phase support. Acta Obstet Gynecol Scand. 2001;80:574-82.
54. MA Lan， Chan Y， Chen JS. The Influence of Pregnancy Outcomes with Estradiol Valerate in Addition during Luteal Phase in IVF-ET Cycles. Journal of Kunming Medical University. 2013; 11:93-6.
55. Martinez F, Coroleu B, Parera N, Alvarez M, Traver JM, Boada M, Barri PN. Human chorionic gonadotropin and intravaginal natural progesterone are equally effective for luteal phase support in IVF. Gynecol Endocrinol. 2000;14:316-20.
56. Mele D, Caprio F, D'Eufemia MD, Schiattarella A, Labriola D, Schettino MT, Colacurci N. In vitro fertilization and psychological stress: new insight about different routes of progesterone administration. Italian journal of gynaecology and obstetrics. 2020;32:119-25.
57. Michnova L, Dostal J, Kudela M, Hamal P, Langova K. Vaginal use of micronized progesterone for luteal support.A randomized study comparing Utrogestan® and Crinone® 8. Biomedical

papers of the medical faculty of the university palacky Olomouc Czechoslovakia. 2017;161:86–91.

58. Miller CE, Zbella E, Webster BW, Doody KJ, Bush MR, Collins MG. Clinical comparison of ovarian stimulation and luteal support agents in patients undergoing GnRH antagonist IVF cycles. J Reprod Med. 2013;58:153-60
59. Mochtar MH, Van Wely M, Van der Veen F. Timing luteal phase support in GnRH agonist down-regulated IVF/ embryo transfer cycles. Hum Reprod. 2006;21:905-8.
60. Moini A, Modarress SZ, Amirchaghmaghi E, Mirghavam N, Khafri S, Akhoond MR, Yazdi RS. The effect of adding oral oestradiol to progesterone as luteal phase support in ART cycles - a randomized controlled study. Arch Med Sci. 2011;7:112-6.
61. Moini A, Zafarani F, Eslami B, Sadeghi M, Kamyabi Z, Jahangiri N. Comparing intramuscular progesterone, vaginal progesterone and 17 alpha-Hydroxyprogestrone caproate in IVF and ICSI cycle. Iran J Reprod Med. 2011;9:119-24.
62. Mona MBR, Shahinaz HE, Tarek MEE, Manal MA. Does Supporting the Luteal Phase With Progesterone Only Without Estradiol Can Affect the Pregnancy Outcome in In-Vitro Fertilization Cycles? Med J Cairo Univ. 2019;87: 4109-14.
63. Mui Lam P, Chun Cheung M, Ping Cheung L, Ingrid Lok H, John Haines C. Effects of early luteal-phase vaginal progesterone supplementation on the outcome of in vitro fertilization and embryo transfer. Gynecol Endocrinol. 2008;24:674-80.
64. Ng EHY, Miao B, Cheung W, Ho PC. A randomized comparison of side effects and patient inconvenience of two vaginal progesterone formulations used for luteal support in in vitro fertilisation cycles. Eur J Obstet Gynecol Reprod Biol. 2003;111:50-4.
65. O Soon N, Sang Hoon L, Do Whan B. A Comparative Study on Clinical Effectiveness of Human Chorionic Gonadotropin and Progesterone on Luteal Support in Controlled Ovarian Hyperstimulatian far IVF. Korea Journal of Fertility and Sterility. 1994;21:233-40
66. Patki A, Pawar VC. Modulating fertility outcome in assisted reproductive technologies by the use of dydrogesterone. Gynecol Endocrinol 2007;23 Suppl 1:68-72.
67. Perino M, Brigandi A, Abate FG, Costabile L, Balzano E, Abate A. Intramuscular versus vaginal progesterone in assisted reproduction: a comparative study. Clin Exp Obstet Gynecol. 1997;24:228-31.

68. Pouly JL, Bassil S, Frydman R, Hedon B, Nicollet B, Prada Y, Antoine JM, Zambrano R, Donnez J. Luteal support after in-vitro fertilization: Crinone 8%, a sustained release vaginal progesterone gel, versus Utrogestan, an oral micronized progesterone. Hum Reprod. 1996;11:2085-9.
69. Propst AM, Hill JA, Ginsburg ES, Hurwitz S, Politch J, Yanushpolsky EH. A randomized study comparing Crinone 8% and intramuscular progesterone supplementation in in vitro fertilization-embryo transfer cycles. Fertil Steril. 2001;76:1144-9.
70. Qublan H, Amarin Z, Al-Quda M, Diab F, Nawasreh M, Malkawi S, Balawneh M. Luteal phase support with GnRH-a improves implantation and pregnancy rates in IVF cycles with endometrium of less-than or equal to 7 mm on day of egg retrieval. Hum Fertil. 2008;11:43-7.
71. Razieh DF, Maryam AR, Nasim T. Beneficial effect of luteal-phase gonadotropin-releasing hormone agonist administration on implantation rate after intracytoplasmic sperm injection. Taiwan J Obstet Gynecol. 2009;48:245-8.
72. Saharkhiz N, Zamaniyan M, Salehpour S, Zadehmodarres SH, Hoseini S, Cheraghi L, Seif S, Baheiraei, N. A comparative study of dydrogesterone and micronized progesterone for luteal phase support during in vitro fertilization (IVF) cycles. Int J Reprod Biomed. 2017;15:45.
73. Saharkhiz N, Salehpour S, Hosseini S, Hosseinirad H, Nazari L. Effects of gonadotropin-releasing hormone agonist (GnRH-a) as luteal phase support in intracytoplasmic sperm injection (ICSI) cycles: a randomized controlled trial. Middle East Fertility Society Journal. 2020;25.
74. Salehpour S, Tamimi M, Saharkhiz N. Comparison of oral dydrogesterone with suppository vaginal progesterone for luteal-phase support in in vitro fertilization (IVF): a randomized clinical trial. Iran J Reprod Med. 2013;11:913-8.
75. Saunders H, Khan C, D'Hooghe T, Magnúsdóttir TB, Klingmann I, Hrafnsdóttir S. vaginal progesterone luteal phase support post IVF study group. Efficacy, safety and tolerability of progesterone vaginal pessaries versus progesterone vaginal gel for luteal phase support after in vitro fertilisation: a randomised controlled trial. Hum Reprod. 2020;35:355-63.
76. Serna J, Cholquevilque JL, Cela V, Martínez-Salazar J, Requena A, Garcia-Velasco JA. Estradiol supplementation during the luteal phase of IVF-ICSI patients: a randomized, controlled trial. Fertil Steril. 2008;90:2190-5.

77. Stadtmauer L, Silverberg KM, Ginsburg ES, Weiss H, Howard B. Progesterone vaginal ring versus vaginal gel for luteal support with in vitro fertilization: a randomized comparative study. Fertil Steril. 2013;99:1543-9.

78. Tay PY, Lenton EA. Inhibition of progesterone secretion by oestradiol administered in the luteal phase of assisted conception cycles. Med J Malaysia. 2003;58:187-95.

79. Tay PYS, Lenton EA. The impact of luteal supplement on pregnancy outcome following stimulated IVF cycles. Med J Malaysia. 2005;60:151-7.

80. Tomic V, Tomic J, Klaic DZ, Kasum M, Kuna K. Oral dydrogesterone versus vaginal progesterone gel in the luteal phase support: randomized controlled trial. Eur J Obstet Gynecol Reprod Biol. 2015;186:49-53.

81. Torode HW, Porter RN, Vaughan JI, Saunders DM. Luteal phase support after in vitro fertilisation: a trial and rationale for selective use. Clin Reprod Fertil. 1987;5:255-61.

82. Tournaye H, Sukhikh GT, Kahler E, Griesinger G. A Phase III randomized controlled trial comparing the efficacy, safety and tolerability of oral dydrogesterone versus micronized vaginal progesterone for luteal support in in vitro fertilization. Hum Reprod. 2017;32:1019−27.

83. Wang XX, Yi XF, Fang YY, Zhao DN, Ye YX. The influence of pregnancy outcomes with oestradiol valeratein addition during luteal phase in IVF-ET cycles. Chinese Journal of Practical Gynecology and Obstetrics. 2009;25:938-40.

84. Wong YF, Loong EPL, Mao KR, Tam PPL, Panesar NS, N E, Chang AMZ. Salivary oestradiol and progesterone after in vitro fertilization and embryo transfer using different luteal support regimens. Reprod Fertil Dev. 1990;2:351-8.

85. Yanushpolsky E, Hurwitz S, Greenberg L, Racowsky C, Hornstein M. Crinone vaginal gel is equally effective and better tolerated than intramuscular progesterone for luteal phase support in in vitro fertilization-embryo transfer cycles: a prospective randomized study. Fertil Steril. 2010;94:2596-9.

86. Zafardoust S, Jeddi-Tehrani M, Akhondi MM, Sadeghi MR, Kamali K, Mokhtar S, Badehnoosh B, Arjmand-Teymouri F, Fatemi F, Mohammadzadeh A. Effect of Administration of Single Dose GnRH Agonist in Luteal Phase on Outcome of ICSI-ET Cycles in Women with Previous History of IVF/ICSI Failure: A Randomized Controlled Trial. J Reprod Infertil. 2015;16:96-101.

87. Zargar M, Saadati N, Ejtahed MS. Comparison the effectiveness of oral dydrogesterone, vaginal progesterone suppository and progesterone ampule for luteal phase support on pregnancy rate during ART cycles. International journal of pharmaceutical research and allied sciences. 2016;5:229–36.

88. Zegers-Hochschild F, Balmaceda JP, Fabres C, Alam V, Mackenna A, Fernández E, Pacheco IM, Sepúlveda MS, Chen S, Borrero C, et al. Prospective randomized trial to evaluate the efficacy of a vaginal ring releasing progesterone for IVF and oocyte donation. Hum Reprod. 2000;15:2093-7.

89. Zhang H, Wang W, Ma W, Cai Q, Liu Y. The influence of pregnancy outcomes with oestradiol valerate in addition to progesterone for luteal supplementation in IVF-ET cycles. Matern Child Health Care China. 2008;29:4163-5.

90. *Fujiwara T. A multi-center, randomized, open-label, parallel group study of a natural micronized progesterone vaginal tablet as a luteal support agent in Japanese women undergoing assisted reproductive technology. Reprod Med Biol. 2015;14:185-93.

91. * Ng EHY, Chan CCW, Tang OS, Ho PC. A randomized comparison of side effects and patient convenience between Cyclogest suppositories and Endometrin tablets used for luteal phase support in IVF treatment. Eur J Obstet Gynecol Reprod Biol. 2007;131:182-8.

92. # Iwase A, Ando H, Toda S, Ishimatsu S, Harata T, Kurotsuchi S, Shimomura Y, Goto M, Kikkawa F. Oral progestogen versus intramuscular progesterone for luteal support after assisted reproductive technology treatment: a prospective randomized study. Arch Gynecol Obstet. 2008;277:319-24.

93. # Ludwig M, Schwartz P, Babahan B, Katalinic A, Weiss JM, Felberbaum R, Al-Hasani S, Diedrich K. Luteal phase support using either Crinone 8% or Utrogest: results of a prospective, randomized study. Eur J Obstet Gynecol Reprod Biol. 2002;103:48-52.

94. ‡ Gao J, Gu F, Miao BY, Chen MH, Zhou CQ, Xu YW. Effect of the initiation of progesterone supplementation in in vitro fertilization–embryo transfer outcomes: a prospective randomized controlled trial. Fertil Steril. 2018;109:97-103.

95. ‡ Williams SC, Oehninger S, Gibbons WE, Van Cleave WC, Muasher SJ. Delaying the initiation of progesterone supplementation results in decreased pregnancy rates after in vitro fertilization: a randomized, prospective study. Fertil Steril. 2001;76:1140-3.

96. Tonguc E, Var T, Ozyer S, Citil A, Dogan M. Estradiol supplementation during the luteal phase of in vitro fertilization cycles: a prospective randomised study. Eur J Obstet Gynecol Reprod Biol. 2011;154:172-6.

---

* Studies 90-91 were excluded comparing vaginal tablet with vaginal suppository or different doses of progesterone.

# Studies 92-93 were excluded using extra luteal phase support after conforming pregnant in both groups.

‡ Studies 94-95 were excluded because they could not be classified.

Study 96 was excluded due to similar luteal phase support in groups.
