# Supplementary figures and images for: Luteal phase support for in vitro fertilization/intracytoplasmic sperm injection fresh cycles: a systematic review and network meta-analysis
Source: Reprod Biol Endocrinol. 2021 Jul 6;19:103. doi: 10.1186/s12958-021-00782-5 (PMC8259396; doi:10.1186/s12958-021-00782-5)

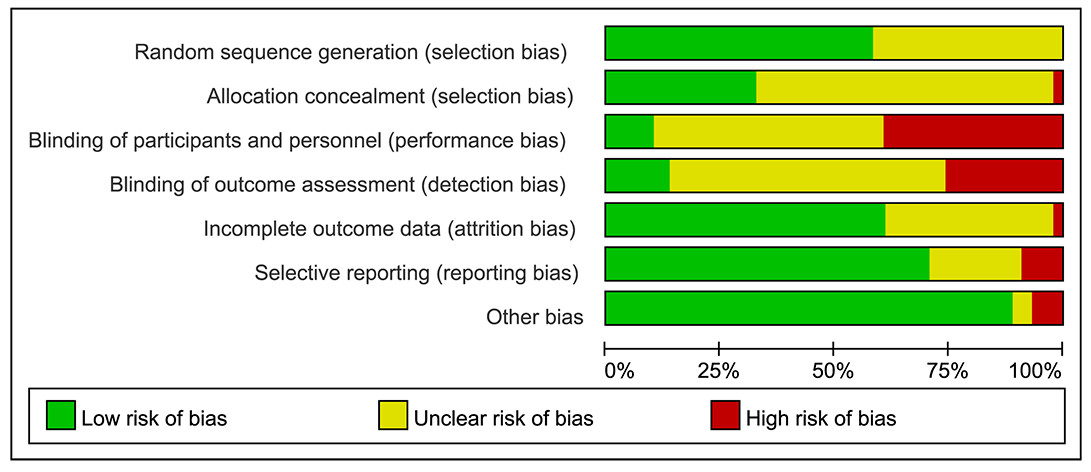

Supplement: Supplementary file 2 — Additional file 2: Figure S1. Risk of bias presented as percentages across all included studies. Figure S2. Summary of risk of bias for each trial. Figure S3. Network meta-analysis (NMA) of clinical pregnancy and miscarriage according to luteal phase support initiation strategy. Abbreviations: HCG=human chorionic gonadotrophin; VPP=vaginal progesterone pessary; VPG=vaginal progesterone gel; IMP=intramuscular progesterone; OP=oral progesterone; RP=rectal progesterone. Figure S4. Global and local inconsistency tests between direct and indirect estimates in the analyses of the pregnancy outcomes. Blue alphabets represent early luteal phase supports. Yellow alphabets represent delayed luteal phase supports. A=placebo; B=human chorionic gonadotrophin; C=vaginal progesterone pessary; D=vaginal progesterone gel; E=intramuscular progesterone; F=oral progesterone; G=rectal progesterone. Figure S5. Network meta-analysis (NMA) for clinical pregnancy and miscarriage. Figure S6. The effects of adding oestrogen, HCG or GnRH agonists in the luteal phase on the pregnancy outcomes. Abbreviations: GnRH, gonadotropin releasing hormone; HCG, human chorionic gonadotrophin. Figure S7. The effects of luteal phase support on adverse events. Figure S8. Global and local inconsistency tests between direct and indirect estimates in the analyses of the pregnancy outcomes (regardless of the initiations of luteal phase supports). A=placebo; B=human chorionic gonadotrophin; C=vaginal progesterone pessary; D=vaginal progesterone gel; E=intramuscular progesterone; F=oral progesterone; G=rectal progesterone; H=vaginal progesterone pessary+ oestrogen; J=intramuscular progesterone+ oestrogen; K= vaginal progesterone pessary + human chorionic gonadotrophin; L=intramuscular progesterone+ human chorionic gonadotrophin; N=subcutaneous progesterone; O=vaginal progesterone ring. Figure S9. Comparison-adjusted funnel plot for the pregnancy outcomes. Figure S10. Network meta-analysis (NMA) for ongoing pregnanc [file 12958_2021_782_MOESM2_ESM.zip › Figure S1.tif]

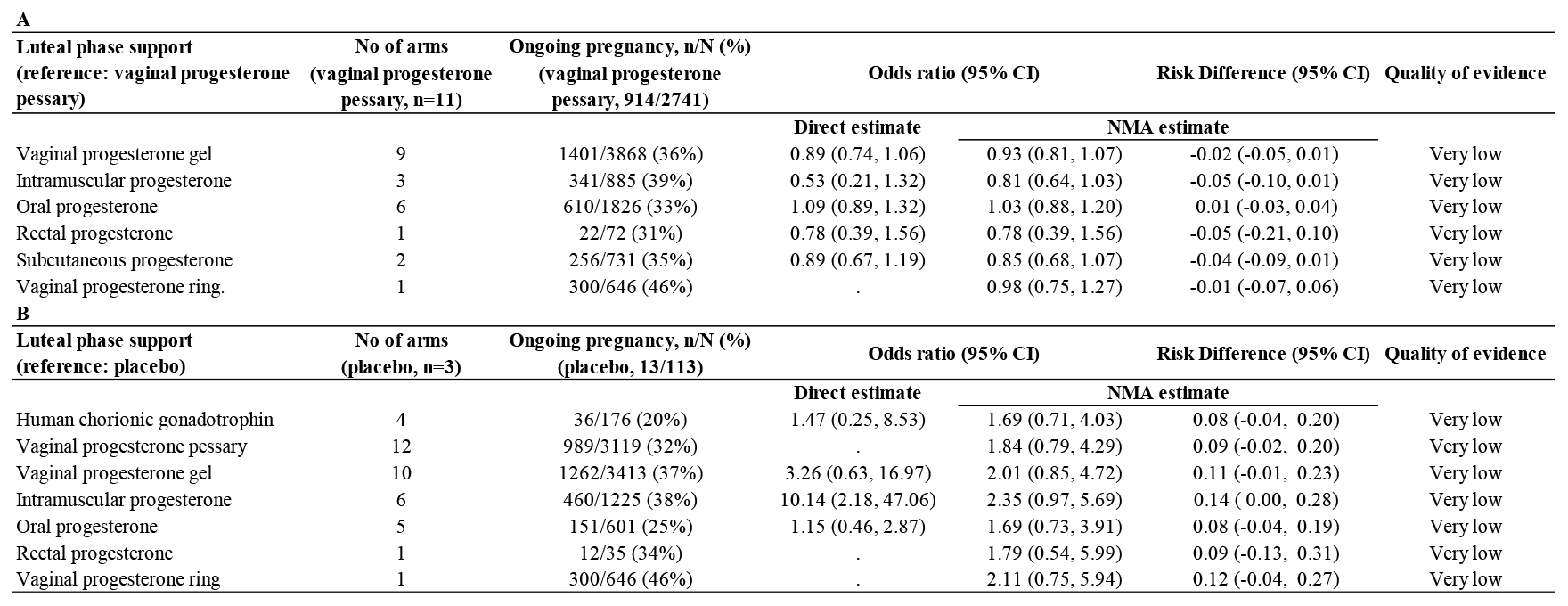

Supplement: Supplementary file 2 — Additional file 2: Figure S1. Risk of bias presented as percentages across all included studies. Figure S2. Summary of risk of bias for each trial. Figure S3. Network meta-analysis (NMA) of clinical pregnancy and miscarriage according to luteal phase support initiation strategy. Abbreviations: HCG=human chorionic gonadotrophin; VPP=vaginal progesterone pessary; VPG=vaginal progesterone gel; IMP=intramuscular progesterone; OP=oral progesterone; RP=rectal progesterone. Figure S4. Global and local inconsistency tests between direct and indirect estimates in the analyses of the pregnancy outcomes. Blue alphabets represent early luteal phase supports. Yellow alphabets represent delayed luteal phase supports. A=placebo; B=human chorionic gonadotrophin; C=vaginal progesterone pessary; D=vaginal progesterone gel; E=intramuscular progesterone; F=oral progesterone; G=rectal progesterone. Figure S5. Network meta-analysis (NMA) for clinical pregnancy and miscarriage. Figure S6. The effects of adding oestrogen, HCG or GnRH agonists in the luteal phase on the pregnancy outcomes. Abbreviations: GnRH, gonadotropin releasing hormone; HCG, human chorionic gonadotrophin. Figure S7. The effects of luteal phase support on adverse events. Figure S8. Global and local inconsistency tests between direct and indirect estimates in the analyses of the pregnancy outcomes (regardless of the initiations of luteal phase supports). A=placebo; B=human chorionic gonadotrophin; C=vaginal progesterone pessary; D=vaginal progesterone gel; E=intramuscular progesterone; F=oral progesterone; G=rectal progesterone; H=vaginal progesterone pessary+ oestrogen; J=intramuscular progesterone+ oestrogen; K= vaginal progesterone pessary + human chorionic gonadotrophin; L=intramuscular progesterone+ human chorionic gonadotrophin; N=subcutaneous progesterone; O=vaginal progesterone ring. Figure S9. Comparison-adjusted funnel plot for the pregnancy outcomes. Figure S10. Network meta-analysis (NMA) for ongoing pregnanc [file 12958_2021_782_MOESM2_ESM.zip › Figure S10.tif]

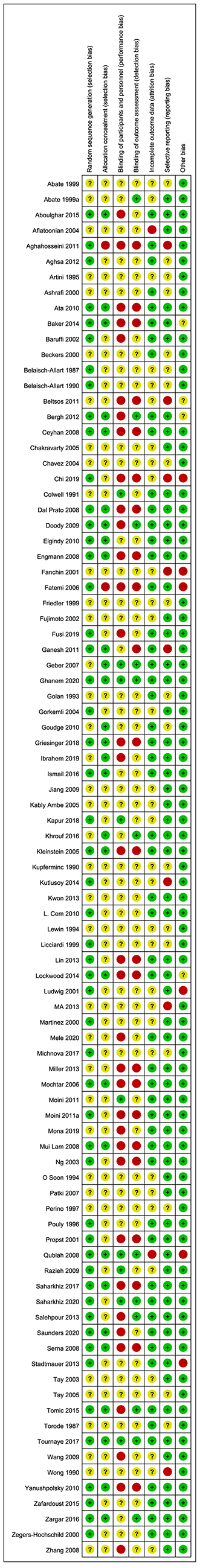

Supplement: Supplementary file 2 — Additional file 2: Figure S1. Risk of bias presented as percentages across all included studies. Figure S2. Summary of risk of bias for each trial. Figure S3. Network meta-analysis (NMA) of clinical pregnancy and miscarriage according to luteal phase support initiation strategy. Abbreviations: HCG=human chorionic gonadotrophin; VPP=vaginal progesterone pessary; VPG=vaginal progesterone gel; IMP=intramuscular progesterone; OP=oral progesterone; RP=rectal progesterone. Figure S4. Global and local inconsistency tests between direct and indirect estimates in the analyses of the pregnancy outcomes. Blue alphabets represent early luteal phase supports. Yellow alphabets represent delayed luteal phase supports. A=placebo; B=human chorionic gonadotrophin; C=vaginal progesterone pessary; D=vaginal progesterone gel; E=intramuscular progesterone; F=oral progesterone; G=rectal progesterone. Figure S5. Network meta-analysis (NMA) for clinical pregnancy and miscarriage. Figure S6. The effects of adding oestrogen, HCG or GnRH agonists in the luteal phase on the pregnancy outcomes. Abbreviations: GnRH, gonadotropin releasing hormone; HCG, human chorionic gonadotrophin. Figure S7. The effects of luteal phase support on adverse events. Figure S8. Global and local inconsistency tests between direct and indirect estimates in the analyses of the pregnancy outcomes (regardless of the initiations of luteal phase supports). A=placebo; B=human chorionic gonadotrophin; C=vaginal progesterone pessary; D=vaginal progesterone gel; E=intramuscular progesterone; F=oral progesterone; G=rectal progesterone; H=vaginal progesterone pessary+ oestrogen; J=intramuscular progesterone+ oestrogen; K= vaginal progesterone pessary + human chorionic gonadotrophin; L=intramuscular progesterone+ human chorionic gonadotrophin; N=subcutaneous progesterone; O=vaginal progesterone ring. Figure S9. Comparison-adjusted funnel plot for the pregnancy outcomes. Figure S10. Network meta-analysis (NMA) for ongoing pregnanc [file 12958_2021_782_MOESM2_ESM.zip › Figure S2.tif]

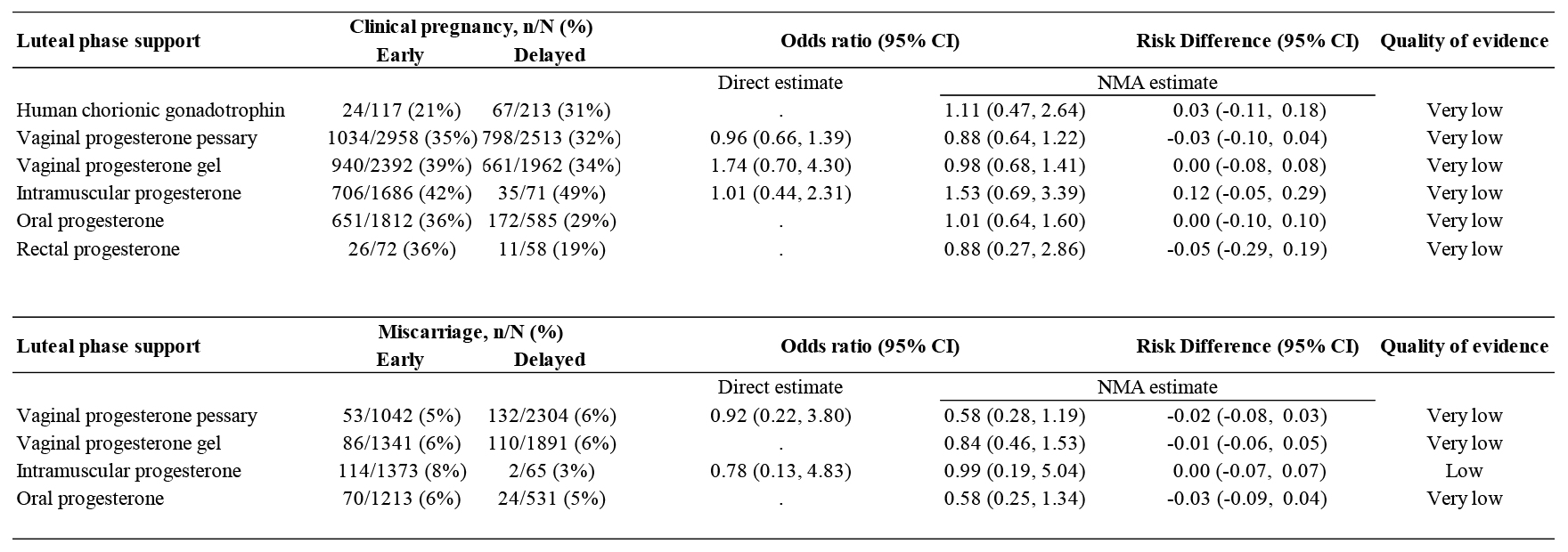

Supplement: Supplementary file 2 — Additional file 2: Figure S1. Risk of bias presented as percentages across all included studies. Figure S2. Summary of risk of bias for each trial. Figure S3. Network meta-analysis (NMA) of clinical pregnancy and miscarriage according to luteal phase support initiation strategy. Abbreviations: HCG=human chorionic gonadotrophin; VPP=vaginal progesterone pessary; VPG=vaginal progesterone gel; IMP=intramuscular progesterone; OP=oral progesterone; RP=rectal progesterone. Figure S4. Global and local inconsistency tests between direct and indirect estimates in the analyses of the pregnancy outcomes. Blue alphabets represent early luteal phase supports. Yellow alphabets represent delayed luteal phase supports. A=placebo; B=human chorionic gonadotrophin; C=vaginal progesterone pessary; D=vaginal progesterone gel; E=intramuscular progesterone; F=oral progesterone; G=rectal progesterone. Figure S5. Network meta-analysis (NMA) for clinical pregnancy and miscarriage. Figure S6. The effects of adding oestrogen, HCG or GnRH agonists in the luteal phase on the pregnancy outcomes. Abbreviations: GnRH, gonadotropin releasing hormone; HCG, human chorionic gonadotrophin. Figure S7. The effects of luteal phase support on adverse events. Figure S8. Global and local inconsistency tests between direct and indirect estimates in the analyses of the pregnancy outcomes (regardless of the initiations of luteal phase supports). A=placebo; B=human chorionic gonadotrophin; C=vaginal progesterone pessary; D=vaginal progesterone gel; E=intramuscular progesterone; F=oral progesterone; G=rectal progesterone; H=vaginal progesterone pessary+ oestrogen; J=intramuscular progesterone+ oestrogen; K= vaginal progesterone pessary + human chorionic gonadotrophin; L=intramuscular progesterone+ human chorionic gonadotrophin; N=subcutaneous progesterone; O=vaginal progesterone ring. Figure S9. Comparison-adjusted funnel plot for the pregnancy outcomes. Figure S10. Network meta-analysis (NMA) for ongoing pregnanc [file 12958_2021_782_MOESM2_ESM.zip › Figure S3.tif]

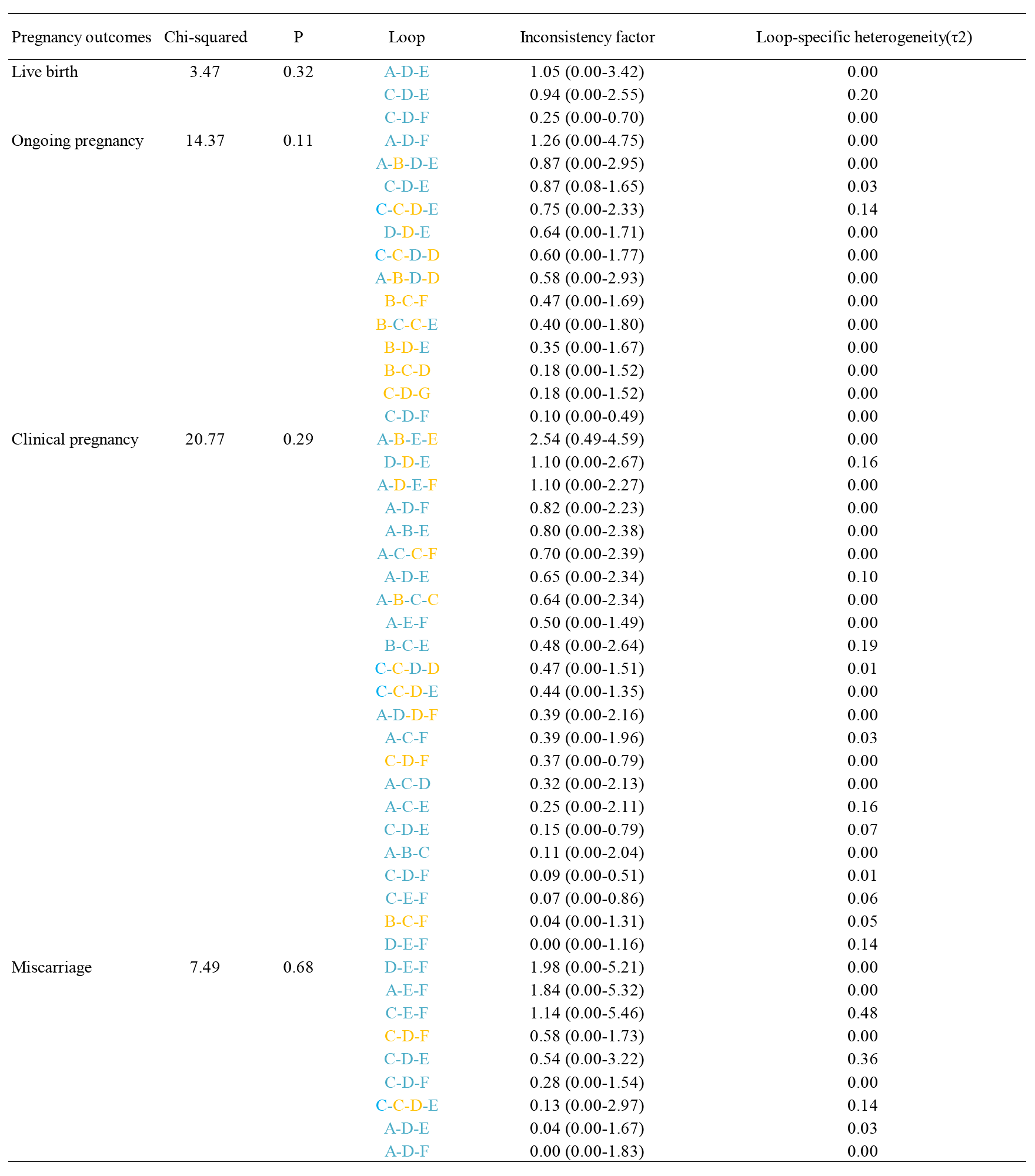

Supplement: Supplementary file 2 — Additional file 2: Figure S1. Risk of bias presented as percentages across all included studies. Figure S2. Summary of risk of bias for each trial. Figure S3. Network meta-analysis (NMA) of clinical pregnancy and miscarriage according to luteal phase support initiation strategy. Abbreviations: HCG=human chorionic gonadotrophin; VPP=vaginal progesterone pessary; VPG=vaginal progesterone gel; IMP=intramuscular progesterone; OP=oral progesterone; RP=rectal progesterone. Figure S4. Global and local inconsistency tests between direct and indirect estimates in the analyses of the pregnancy outcomes. Blue alphabets represent early luteal phase supports. Yellow alphabets represent delayed luteal phase supports. A=placebo; B=human chorionic gonadotrophin; C=vaginal progesterone pessary; D=vaginal progesterone gel; E=intramuscular progesterone; F=oral progesterone; G=rectal progesterone. Figure S5. Network meta-analysis (NMA) for clinical pregnancy and miscarriage. Figure S6. The effects of adding oestrogen, HCG or GnRH agonists in the luteal phase on the pregnancy outcomes. Abbreviations: GnRH, gonadotropin releasing hormone; HCG, human chorionic gonadotrophin. Figure S7. The effects of luteal phase support on adverse events. Figure S8. Global and local inconsistency tests between direct and indirect estimates in the analyses of the pregnancy outcomes (regardless of the initiations of luteal phase supports). A=placebo; B=human chorionic gonadotrophin; C=vaginal progesterone pessary; D=vaginal progesterone gel; E=intramuscular progesterone; F=oral progesterone; G=rectal progesterone; H=vaginal progesterone pessary+ oestrogen; J=intramuscular progesterone+ oestrogen; K= vaginal progesterone pessary + human chorionic gonadotrophin; L=intramuscular progesterone+ human chorionic gonadotrophin; N=subcutaneous progesterone; O=vaginal progesterone ring. Figure S9. Comparison-adjusted funnel plot for the pregnancy outcomes. Figure S10. Network meta-analysis (NMA) for ongoing pregnanc [file 12958_2021_782_MOESM2_ESM.zip › Figure S4.tif]

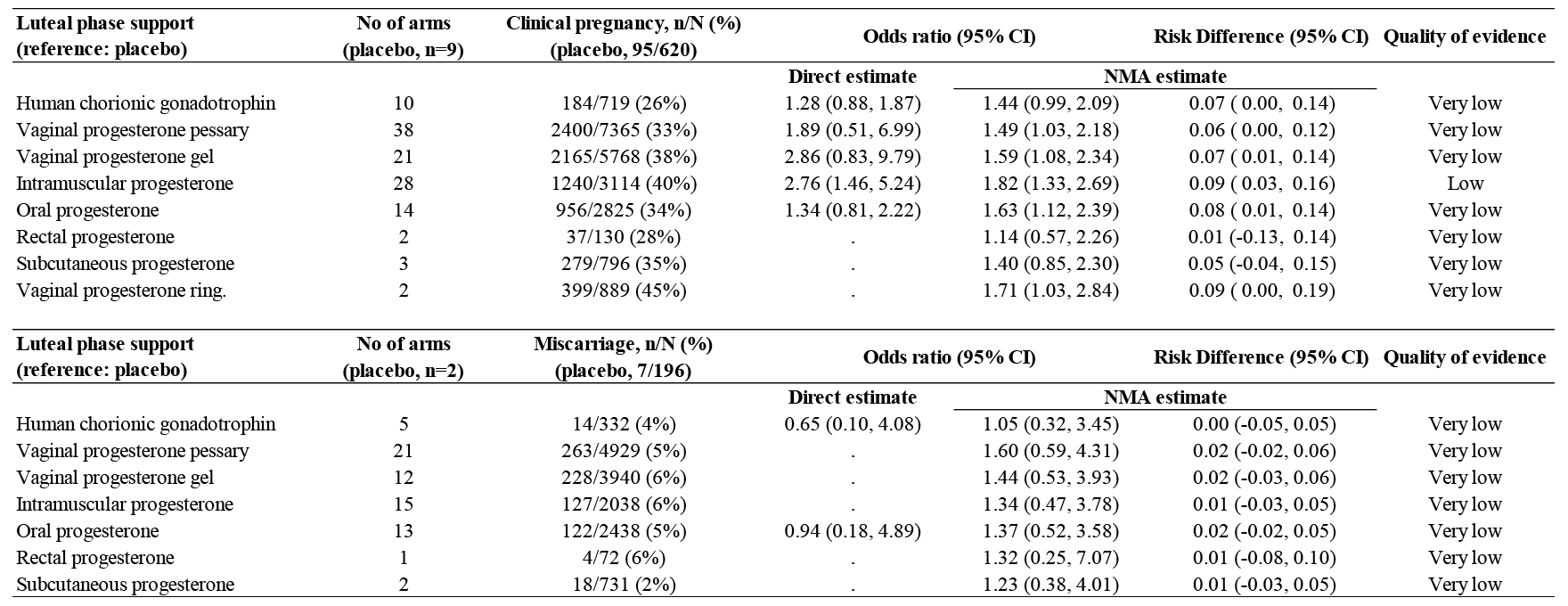

Supplement: Supplementary file 2 — Additional file 2: Figure S1. Risk of bias presented as percentages across all included studies. Figure S2. Summary of risk of bias for each trial. Figure S3. Network meta-analysis (NMA) of clinical pregnancy and miscarriage according to luteal phase support initiation strategy. Abbreviations: HCG=human chorionic gonadotrophin; VPP=vaginal progesterone pessary; VPG=vaginal progesterone gel; IMP=intramuscular progesterone; OP=oral progesterone; RP=rectal progesterone. Figure S4. Global and local inconsistency tests between direct and indirect estimates in the analyses of the pregnancy outcomes. Blue alphabets represent early luteal phase supports. Yellow alphabets represent delayed luteal phase supports. A=placebo; B=human chorionic gonadotrophin; C=vaginal progesterone pessary; D=vaginal progesterone gel; E=intramuscular progesterone; F=oral progesterone; G=rectal progesterone. Figure S5. Network meta-analysis (NMA) for clinical pregnancy and miscarriage. Figure S6. The effects of adding oestrogen, HCG or GnRH agonists in the luteal phase on the pregnancy outcomes. Abbreviations: GnRH, gonadotropin releasing hormone; HCG, human chorionic gonadotrophin. Figure S7. The effects of luteal phase support on adverse events. Figure S8. Global and local inconsistency tests between direct and indirect estimates in the analyses of the pregnancy outcomes (regardless of the initiations of luteal phase supports). A=placebo; B=human chorionic gonadotrophin; C=vaginal progesterone pessary; D=vaginal progesterone gel; E=intramuscular progesterone; F=oral progesterone; G=rectal progesterone; H=vaginal progesterone pessary+ oestrogen; J=intramuscular progesterone+ oestrogen; K= vaginal progesterone pessary + human chorionic gonadotrophin; L=intramuscular progesterone+ human chorionic gonadotrophin; N=subcutaneous progesterone; O=vaginal progesterone ring. Figure S9. Comparison-adjusted funnel plot for the pregnancy outcomes. Figure S10. Network meta-analysis (NMA) for ongoing pregnanc [file 12958_2021_782_MOESM2_ESM.zip › Figure S5.tif]

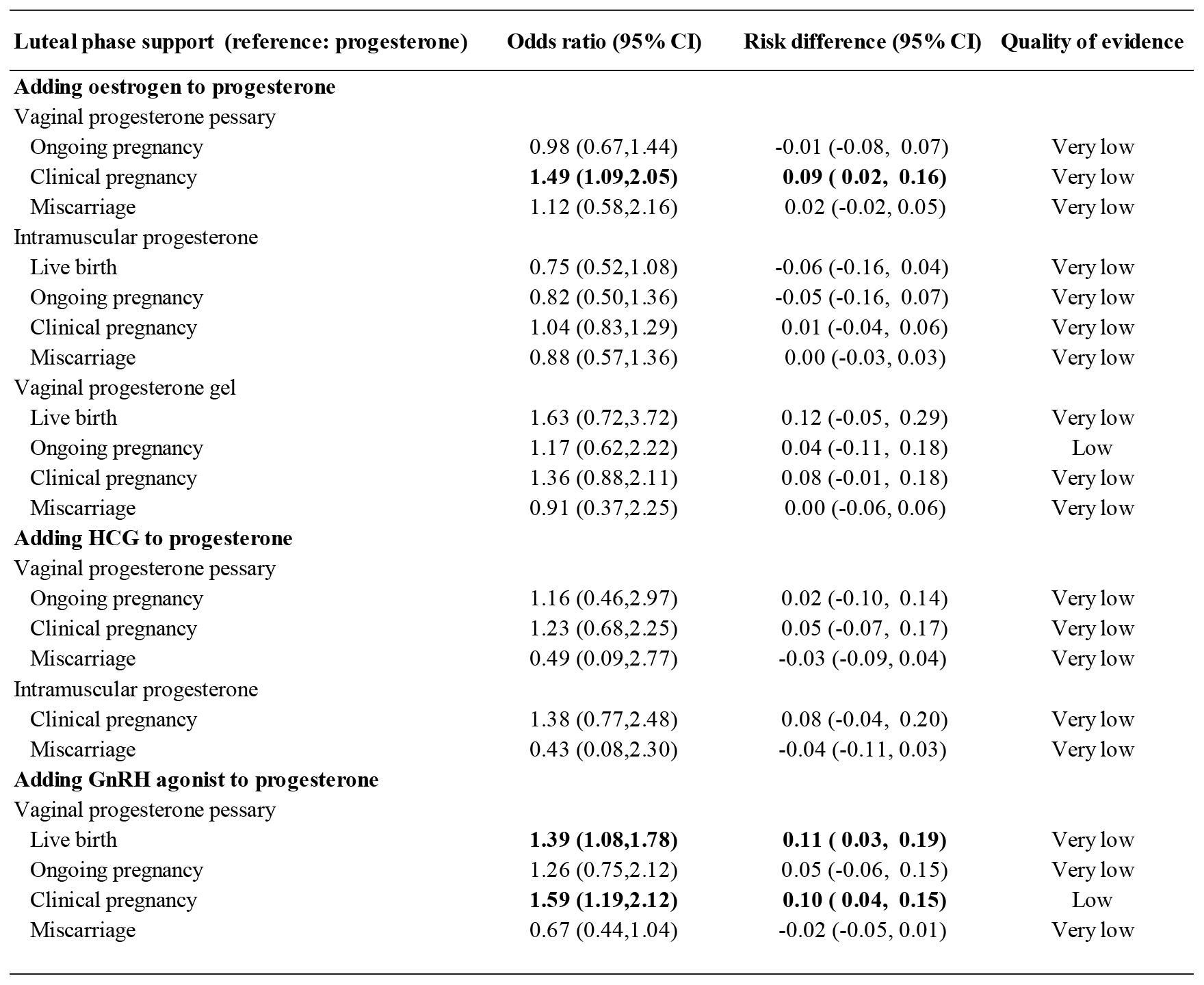

Supplement: Supplementary file 2 — Additional file 2: Figure S1. Risk of bias presented as percentages across all included studies. Figure S2. Summary of risk of bias for each trial. Figure S3. Network meta-analysis (NMA) of clinical pregnancy and miscarriage according to luteal phase support initiation strategy. Abbreviations: HCG=human chorionic gonadotrophin; VPP=vaginal progesterone pessary; VPG=vaginal progesterone gel; IMP=intramuscular progesterone; OP=oral progesterone; RP=rectal progesterone. Figure S4. Global and local inconsistency tests between direct and indirect estimates in the analyses of the pregnancy outcomes. Blue alphabets represent early luteal phase supports. Yellow alphabets represent delayed luteal phase supports. A=placebo; B=human chorionic gonadotrophin; C=vaginal progesterone pessary; D=vaginal progesterone gel; E=intramuscular progesterone; F=oral progesterone; G=rectal progesterone. Figure S5. Network meta-analysis (NMA) for clinical pregnancy and miscarriage. Figure S6. The effects of adding oestrogen, HCG or GnRH agonists in the luteal phase on the pregnancy outcomes. Abbreviations: GnRH, gonadotropin releasing hormone; HCG, human chorionic gonadotrophin. Figure S7. The effects of luteal phase support on adverse events. Figure S8. Global and local inconsistency tests between direct and indirect estimates in the analyses of the pregnancy outcomes (regardless of the initiations of luteal phase supports). A=placebo; B=human chorionic gonadotrophin; C=vaginal progesterone pessary; D=vaginal progesterone gel; E=intramuscular progesterone; F=oral progesterone; G=rectal progesterone; H=vaginal progesterone pessary+ oestrogen; J=intramuscular progesterone+ oestrogen; K= vaginal progesterone pessary + human chorionic gonadotrophin; L=intramuscular progesterone+ human chorionic gonadotrophin; N=subcutaneous progesterone; O=vaginal progesterone ring. Figure S9. Comparison-adjusted funnel plot for the pregnancy outcomes. Figure S10. Network meta-analysis (NMA) for ongoing pregnanc [file 12958_2021_782_MOESM2_ESM.zip › Figure S6.tif]

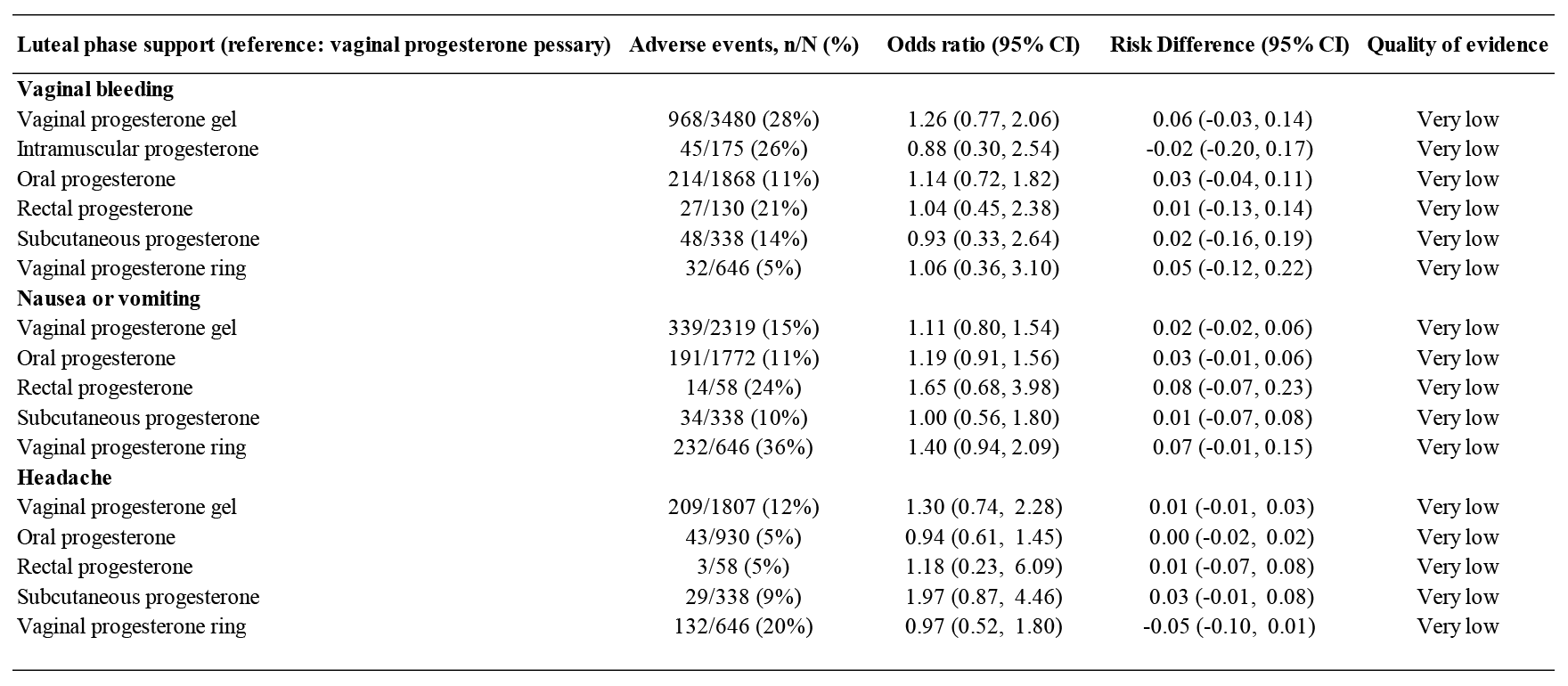

Supplement: Supplementary file 2 — Additional file 2: Figure S1. Risk of bias presented as percentages across all included studies. Figure S2. Summary of risk of bias for each trial. Figure S3. Network meta-analysis (NMA) of clinical pregnancy and miscarriage according to luteal phase support initiation strategy. Abbreviations: HCG=human chorionic gonadotrophin; VPP=vaginal progesterone pessary; VPG=vaginal progesterone gel; IMP=intramuscular progesterone; OP=oral progesterone; RP=rectal progesterone. Figure S4. Global and local inconsistency tests between direct and indirect estimates in the analyses of the pregnancy outcomes. Blue alphabets represent early luteal phase supports. Yellow alphabets represent delayed luteal phase supports. A=placebo; B=human chorionic gonadotrophin; C=vaginal progesterone pessary; D=vaginal progesterone gel; E=intramuscular progesterone; F=oral progesterone; G=rectal progesterone. Figure S5. Network meta-analysis (NMA) for clinical pregnancy and miscarriage. Figure S6. The effects of adding oestrogen, HCG or GnRH agonists in the luteal phase on the pregnancy outcomes. Abbreviations: GnRH, gonadotropin releasing hormone; HCG, human chorionic gonadotrophin. Figure S7. The effects of luteal phase support on adverse events. Figure S8. Global and local inconsistency tests between direct and indirect estimates in the analyses of the pregnancy outcomes (regardless of the initiations of luteal phase supports). A=placebo; B=human chorionic gonadotrophin; C=vaginal progesterone pessary; D=vaginal progesterone gel; E=intramuscular progesterone; F=oral progesterone; G=rectal progesterone; H=vaginal progesterone pessary+ oestrogen; J=intramuscular progesterone+ oestrogen; K= vaginal progesterone pessary + human chorionic gonadotrophin; L=intramuscular progesterone+ human chorionic gonadotrophin; N=subcutaneous progesterone; O=vaginal progesterone ring. Figure S9. Comparison-adjusted funnel plot for the pregnancy outcomes. Figure S10. Network meta-analysis (NMA) for ongoing pregnanc [file 12958_2021_782_MOESM2_ESM.zip › Figure S7.tif]
